# New insights on unspecific peroxygenases: superfamily reclassification and evolution

**DOI:** 10.1186/s12862-019-1394-3

**Published:** 2019-03-13

**Authors:** Muniba Faiza, Shengfeng Huang, Dongming Lan, Yonghua Wang

**Affiliations:** 1 School of Food Science and Engineering, South China University of Technology, Guangzhou, 510640 China; 2 Laboratory for Marine Biology and Biotechnology, Qingdao National Laboratory for Marine Science and Technology, Qingdao, China; 3 State Key Laboratory of Biocontrol, School of Life Sciences, Sun Yat-Sen University, Guangzhou, China

**Keywords:** Fungal genomes, Unspecific peroxygenases, *Agrocybe aegerita*, Chloroperoxidase, Phylogeny, Selection pressure, Functional divergence, Subfamilies, Superfamily

## Abstract

**Background:**

Unspecific peroxygenases (UPO) (EC 1.11.2.1) represent an intriguing oxidoreductase sub-subclass of heme proteins with peroxygenase and peroxidase activity. With over 300 identified substrates, UPOs catalyze numerous oxidations including 1- or 2- electron oxygenation, selective oxyfunctionalizations, which make them most significant in organic syntheses and potentially attractive as industrial biocatalysts. There are very few UPOs available with distinct properties, notably, *Mro*UPO which shows behavior ranging between UPO and another heme-thiolate peroxidase, called Chloroperoxidase (CPO). It prompted us to search for more UPOs in fungal kingdom which led us to studying their relationship with CPO.

**Results:**

In this study, we searched for novel UPOs in more than 800 fungal genomes and found 113 putative UPO-encoding sequences distributed in 35 different fungal species (or strains), amongst which single sequence per species were subjected to phylogeny study along with CPOs. Our phylogenetic study show that the UPOs are distributed in Basidiomycota and Ascomycota phyla of fungi. The sequence analysis helped to classify the UPOs into five distinct subfamilies: classic *Aae*UPO and four new subfamilies with potential new traits. We have also shown that each of these five subfamilies (supported by) have their own signature motifs. Surprisingly, some of the CPOs appeared to be a type of UPOs indicating that they were previously identified incorrectly. Selection pressure was observed on important motifs in UPOs which could have driven their functional divergence. Furthermore, the sites having different evolutionary rates caused by the functional divergence were also identified on some motifs along with the other relevant amino acid residues. Finally, we predicted critical amino acids responsible for the functional divergence in the UPOs and identified some sequence differences among UPOs, CPOs, and *Mro*UPO to predict it’s ranging behavior.

**Conclusion:**

This study discovers new UPOs, provides a glimpse of their evolution from CPOs, and presents new insight on their functional divergence. We present a new classification of UPOs and shed new light on its phylogenetics. These different UPOs may exhibit a wide range of characteristics and specificities which may help in various fields of synthetic chemistry and industrial biocatalysts, and may as well lead to an advancement towards the understanding of physiological role of UPOs in fungi.

**Electronic supplementary material:**

The online version of this article (10.1186/s12862-019-1394-3) contains supplementary material, which is available to authorized users.

## Introduction

Unspecific peroxygenase (UPO), also known as aromatic peroxygenase (APO), are newly discovered extracellular enzymes which belong to heme-thiolate proteins obtained from fungal species [1]. The first UPO enzyme was discovered in *Agrocybe aegerita* (*Aae*UPO) which belongs to Basidiomycota, commonly known as Black Poplar mushroom [2, 3]. *Aae*UPO is known to catalyze a number of reactions leading to the formation of alcohol by transfer of an oxygen atom by reacting with hydrogen peroxide [4–6]. Fungal UPOs are characterized for catalyzing a large variety of reactions such as epoxidation, hydroxylation, dealkylations, oxidation of aromatic and heterocyclic compounds, organic heteroatoms, inorganic halides, and one- and two- electron oxidations as well [6–8]. They exhibit various useful properties such as high specific activity, catalytic activity, and specificity, catalyze reactions with inexpensive peroxides and cofactors (Mg^2+^), stability and it is water-soluble in nature due to a high degree of glycosylation. Hence, they are considered very intriguing enzymes and also termed as ‘closest to ideal biocatalysts for (sub)-terminal hydroxylation of short-chain and medium-chain alkanes under mild conditions’ [9]. The other known fungal UPOs include *Marasmius rotula* (*Mro*UPO) and *Coprinellus radians* (*Cra*UPO). Like *Aae*UPO, *Mro*UPO and *Cra*UPO also belong to order *Agaricales* (known as “gilled mushrooms”) of Basidiomycota phylum.

Chloroperoxidase (CPO) (EC 1.11.1.10) is a well-known heme-thiolate peroxidase (HTP), which has strong peroxidase activity but unlike UPOs, it falls short on peroxygenating aromatic substrates and strong C-H bonds [10]. UPOs are classified as HTPs due to their characteristic heme ligation by a cysteine and resemblance to CPOs. UPOs are generally present in Dikarya, higher fungi that includes the two phylum: Ascomycota and Basidiomycota of the fungal kingdom as also supported by the results obtained in this study, and found lacking in *Taphrinomycotina*, *Saccharomycotina* involving true yeasts such as *Saccharomyces*, fission yeasts such as *Schizosaccharomyces*, all other fungal species as also reported earlier [11, 12]. Based on their molecular mass and motif patterns, UPOs are classified into two categories: Group-I (short UPO sequences) with an average mass of 29 kDa and Group-II (long UPO sequences) with an average mass of 44 kDa [13]. *Mro*UPO (and interestingly, CPO) belong to Group-I UPOs which are widespread in the whole fungal phyla whereas *Aae*UPO and *Cra*UPO belong to Group-II UPOs which are found only in Ascomycota and Basidiomycota.

**Fig. 1:**
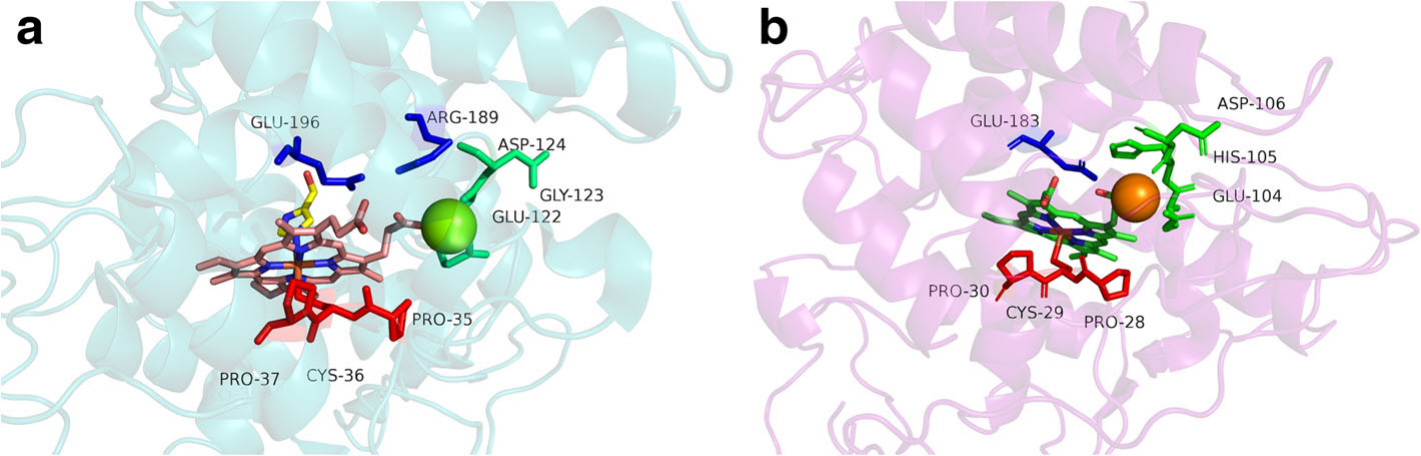
represents the experimentally resolved structures showing conserved motifs in **a**) *Aae*UPO **b**) *Lfu*CPO. The PCP motifs are shown with red sticks, EGD and EHD motifs are shown in green, acid-base catalyst in **a**) and charge stabilizer in **b**) are shown in blue

To date, only two peroxygenase protein crystal structures are available in Protein Data Bank (PDB): *Agrocybe aegerita* (PDB ID: 2YOR) and *Marasmius rotula* (PDB ID: 5FUJ/5FUK), of which the structure of *Aae*UPO solved at 2.2 Å [14] is well studied (Fig. 1). Although as compared to *Aae*UPO, *Mro*UPO shows less peroxygenating activity [15] but it is capable of oxidizing bulkier substrates [16] and produces higher protein yields [15]. The distinctive feature of *Mro*UPO is its limited capability of oxidizing iodide amongst the other halides and hence lacking brominating or chlorinating activities. Although its catalytic behavior towards the peroxidase substrates such as dimethoxyphenol, and peroxygenation of aryl alcohols is similar to the *Aae*UPO and a chloroperoxidase (CPO) namely, *Leptoxyphium fumago* CPO (*Lfu*CPO); and the oxygen transfer potential is slightly higher than that of *Lfu*CPO [1, 17, 18]. Another most relevant catalytic property of *Mro*UPO is its ability to transfer peroxide-borne oxygen to non-activated carbon, on the basis of which its behavior was stated ranging between *Aae*UPO and *Lfu*CPO [15]. These different properties of *Mro*UPO ranging between *Aae*UPO and *Lfu*CPO, suggest its link between both these enzymes. Therefore, we hypothesized that the *Mro*UPO may be bridging a gap between CPOs and UPOs, which despite having motifs similar to CPOs, is capable of functioning as peroxygenase as well. To analyze the relationship between these UPOs and CPOs, we searched for new UPOs in the fungal kingdom.

In order to correctly identify the UPO encoding sequences in fungal genomes, the structural properties and catalytic motifs were thoroughly analyzed in both *Aae*UPO and *Lfu*CPO. The *Aae*UPO has a cone-shaped cavity constituting a binding pocket for substrates, which is surrounded by aromatic residues [14]. Since both UPO and CPO belong to the HTP superfamily, they both have the same PCP (Proline-Cysteine-Proline) motif with the *Cys36* (in *Aae*UPO) and *Cys29* (in *Lfu*CPO) being the proximal ligand for the heme (Fig. 1). This motif is highly conserved in UPOs and required for their catalytic activity [14]. In both the enzymes, the distal heme cavity consists of a negatively charged residue *Glu196* in *Aae*UPO and *Glu183* in CPO. The charge stabilizer in *Aae*UPO is *Arg189* and that in CPO is *His105* [14]. *Arg189-Glu196* acts as the acid-base catalyst pair in *Aae*UPO while in CPO this function is performed by *His105-Glu183*. This acid-base catalyst pair is crucial for the formation of Compound-I in all peroxidases [19, 20] including both CPOs and UPOs during their catalytic process. The *His105* residue is involved in peroxidase function of CPO by participating indirectly in the cleavage of the peroxide bond by forming a hydrogen-bond to direct *Glu183* in the heme center [21]. The third motif required for the catalytic properties of *Aae*UPO is EGD motif (i.e., *Glu122-Gly123-Asp124*), which is slightly different in CPO where *Gly123* present in *Aae*UPO is replaced by *His* in CPO forming EHD motif [14, 22]. Previously, it was also proposed that the *His* residue of EHD motif in CPO is exchanged by the *Gly123* in *Aae*UPO due to which they both do not follow the same mechanism of catalysis [22]. However, there is no strong evidence to support this hypothesis. Since there are not many structural details available about *Mro*UPO, therefore, the motifs were analyzed manually in the sequence of *Mro*UPO revealing the same pattern as exhibited by the CPOs. To summarize, conserved motif patterns for the catalytic activity of *Aae*UPO is -PCP-EGD-R--E, and for *Mro*UPO and CPO is -PCP-EHD-E [7, 22].

Both UPO and CPO are HTPs, both have to undergo the formation of Compound-I in their catalytic processes, and have structural similarities but show different catalytic properties such as CPO is a typical haloperoxidase while UPO displays relatively weaker haloperoxidase activity and predominates at showing peroxygenase property [7]. Therefore, we have used fungal CPOs as the outgroup for the comparison. Since *Mro*UPO exhibits the same motif pattern as possessed by the CPOs, therefore, it was placed among CPOs for further analyses. In this study, we have customized a genome data mining pipeline to search for novel UPOs in 812 fungal genomes available in the Ensembl database.

## Results

### Novel UPO encoding putative sequences in the fungal kingdom

It has been stated that there are thousands of UPO-like sequences present in databases [7] but perhaps these sequences may lack the catalytic motifs. Therefore, both homology search and motif search helped to eliminate the extra sequences which are lacking the required motifs for the catalytic activity of UPO. Finally, we obtained 113 putative UPO sequences from the fungal kingdom which belong to 35 different fungal species including different strains. The largest number of putative sequences come from *Sphaerobolus stellatus ss14* (26 putative sequences) followed by *Galerina marginata cbs339.88* (11 putative sequences), *Agaricus bisporus var burnettii jb137s8* (10 putative sequences), *Exidia glandulosa hhb12029* (9 putative sequences), *Sistotremastrum niveocremeum hhb9708* and *Hypholoma sublaterium fd334ss4* (6 putative sequences), *Coprinopsis cinerea okayama7.130* (5 putative sequences), *Fibulorhizoctonia sp cbs109695* (4 putative sequences), and rest of the species consists of 1–3 putative sequences as shown in (Additional file 1: Table S1). The largest number of putative sequences are gilled mushrooms and come from Agaricomycotina subphylum of Basidiomycota showing high similarity to *Aae*UPO. It shows that most of the putative fungal UPOs reside in Basidiomycota phylum of fungal kingdom.

### Phylogenetic analysis

**Fig. 2:**
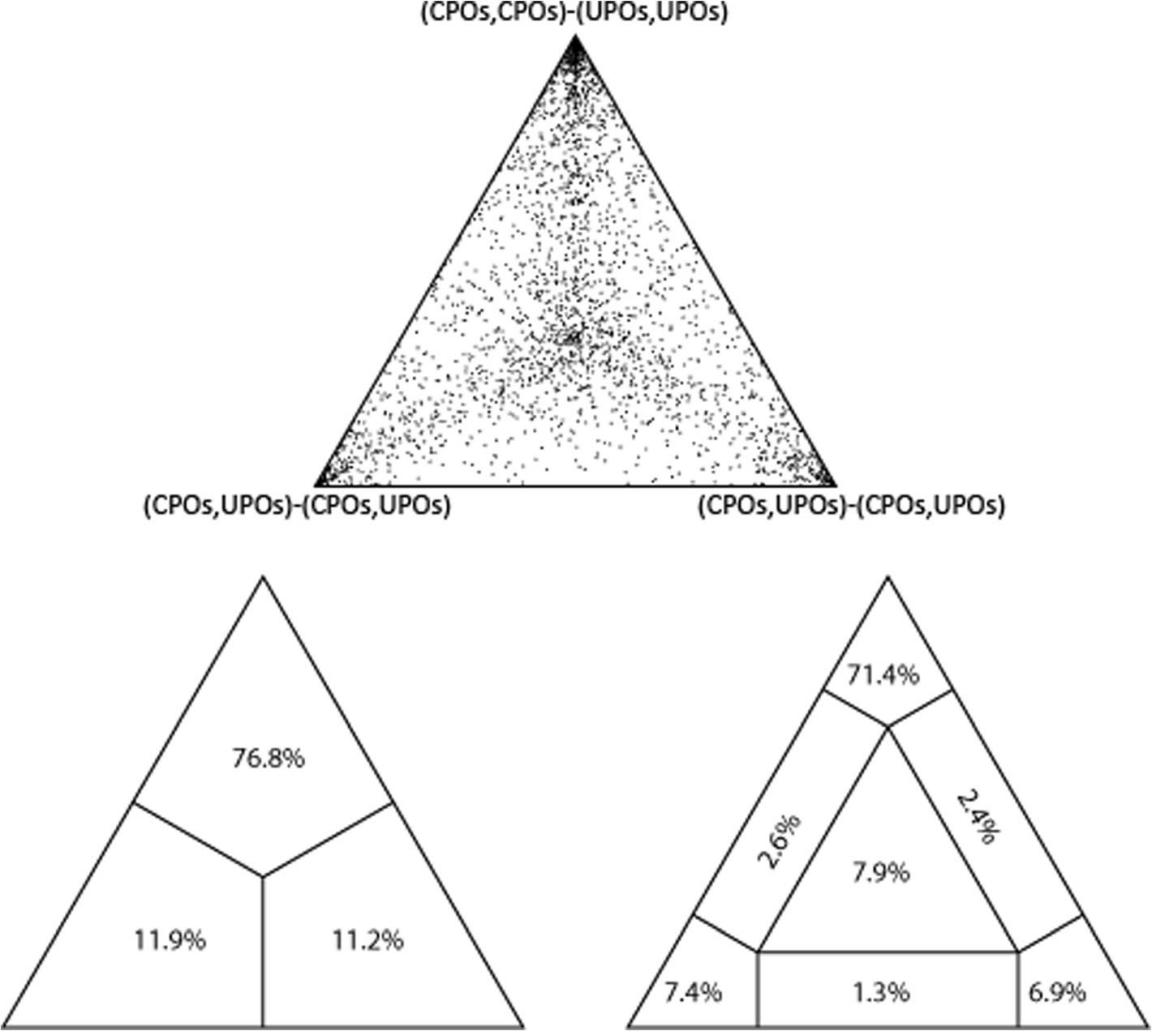
LBA of the constructed phylogeny of CPOs and resultant UPOs showing the completely resolved phylogeny

**Fig. 3:**
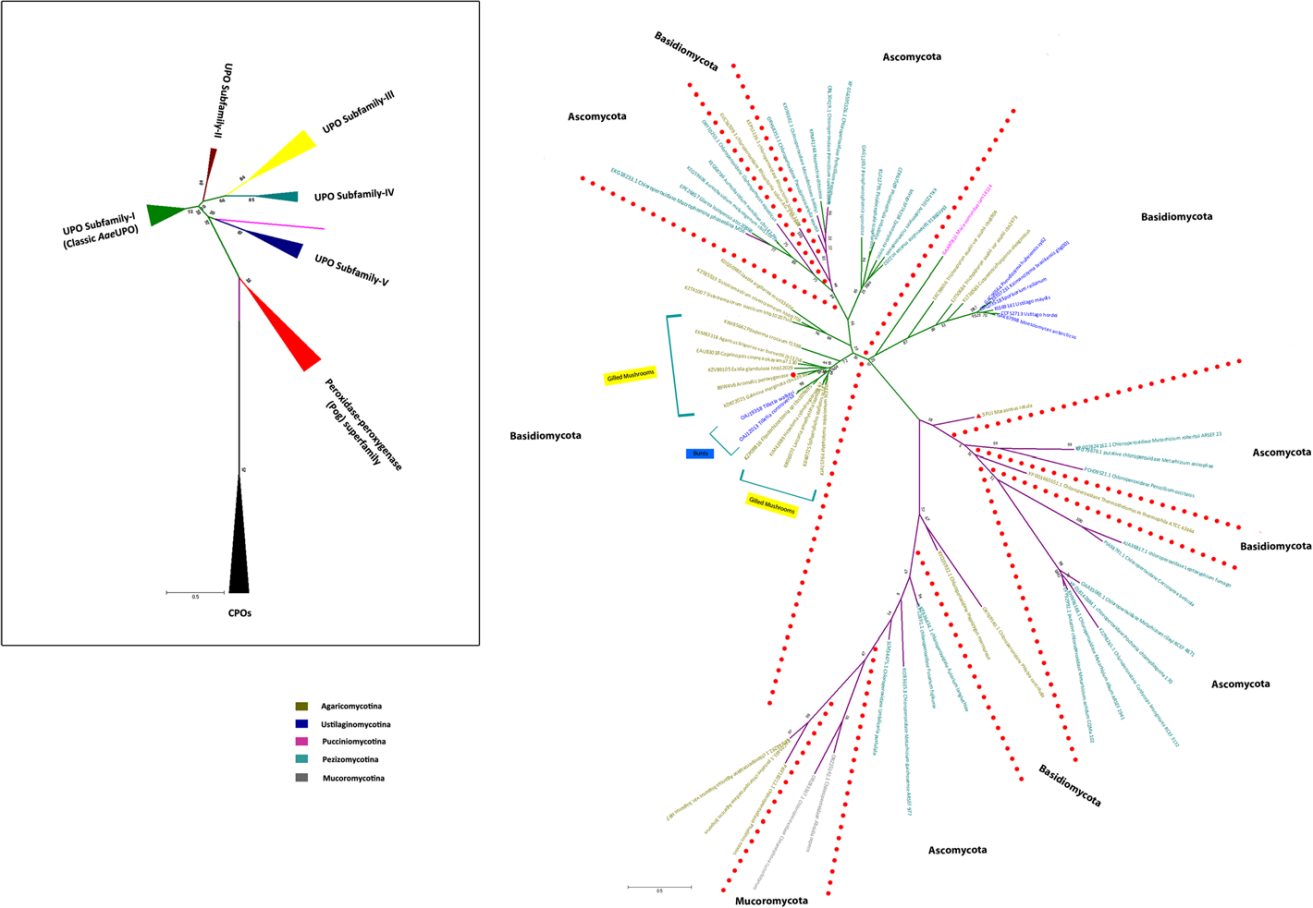
ML tree of resultant 36 newly found UPO sequences (including *Aae*UPO labeled with red dot), 8 CPO sequences now classified as a kind of UPOs, and 23 CPO sequences along with *Mro*UPO sequence (shown with red triangle). Green branches denote UPOs and magenta colored branches denote CPOs. The collapsed tree is shown in the black box representing subfamilies and superfamily of UPOs

The LBA analysis of the constructed phylogeny revealed that the sequences are evolving along a completely resolved phylogeny (Fig. 2) [23]. The proportion of points inside the matrix increases with the length of sequences indicating that the noise caused by sampling artifacts is diminished. The estimated branch lengths of the constructed ML tree were significantly lower showing less amount of change along the sequences (Fig. 3).

The constructed phylogenetic tree of UPO and CPO sequences showed that most of the obtained UPOs belong to the Basidiomycota phylum distributed in three subphyla: Agaricomycotina, Pucciniomycotina, and Ustilaginomycotina. The majority of UPOs belong to Agaricomycotina as it constitutes around 70% of the Basidiomycota followed by Ustilaginomycotina and Pucciniomycotina. Only Pezizomycotina subphylum of Ascomycota was found to be consists of UPO, and found lacking in Wallemiomycotina subphylum of Basidiomycota, and Taphrinomycotina and Saccharomycotina subphyla of Ascomycota. Besides, there was only one fungal genus which consists of both UPO as well as CPO, namely, *Agaricus* which consists of CPO in *Agaricus bisporus* and *Agaricus bisporus var bisporus H97* whereas UPO in *Agaricus bisporus var burnettii jb137s8*, which supports the previously proposed hypothesis that *Agaricus bisporus sp.* may have evolved metabolic strategies and niche adaptations which are lacking in white-rot and brown-rot fungi, and they may also have a distinct distribution of substrate conversion enzymes in adaptation to ecological niche [24]. This could be a possible reason for the presence of both CPO and UPO in *Agaricus bisporus sp.*

Interestingly, some of the CPO sequences are placed among four UPO encoding sequences: *Glarea lozoyensis atcc20868*, *Aureobasidium melanogenum cbs110374*, *Aureobasidium namibiae cbs147.97,* and *Neonectria ditissima* which belong to Pezizomycotina subphylum of Ascomycota suggesting their similar catalytic behavior. The phylogenetic tree shows high scores for these CPO sequences placed among the UPOs. They also have a similar motif pattern as the UPOs with acid-base catalyst required for UPO activity exhibiting EAD/ETD motif in some sequences instead of EGD motif. It is also worth noting that these residues are not defined for CPO activity as well. Besides, there is no report implicating the CPO activity in these sequences which provide a strong evidence that these CPOs may be another kind of UPO mistakenly recognized as CPOs. However, the *Mro*UPO is placed along with the *Lfu*CPO and some other CPO sequences in the phylogenetic tree.

### Conserved motifs in UPOs

**Fig. 4:**
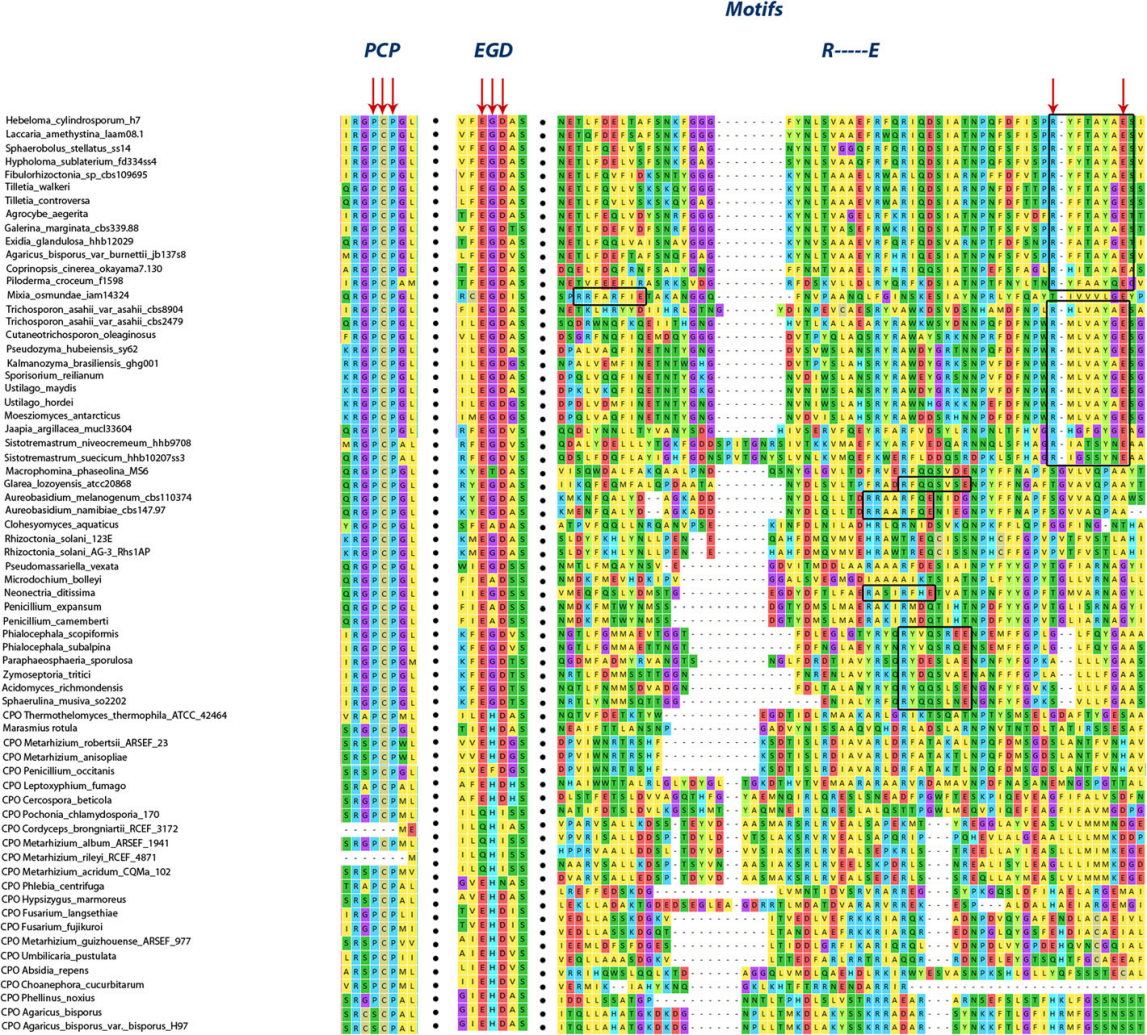
MSA showing the basic previously known motifs present in all resultant UPOs: PCP---EGD---R------E

The multiple sequence alignment (MSA) (see Additional file 2: Figure S1) and structural analysis of all predicted structures revealed that the resultant UPO sequences consist of the motifs (PCP---EGD---R----E) (Fig. 4) required for the enzyme activity and the binding pocket is surrounded by the aromatic amino acid residues (see Additional file 3: Table S2) as exhibited by *Aae*UPO.

Our analysis led to the discovery of two new conserved motifs, which are virtually present in all UPOs, namely, the S [IL] G motif located between the PCP and the EGD motifs and SXXRXD motif present after the EGD motif, except in *Mro*UPO. All UPOs consist *Ile* in S [IL] G motif except three species: *Jaapia argillacea mucl33604, Mixia osmundae iam14324,* and *Sphaerulina musiva so2202,* which contain *Leu* in place of *Ile*. Strangely, three of these species belong to different subphyla, namely Agaricomycotina, Pucciniomycotina, and Pezizomycotina belonging to both Basidiomycota phylum and Ascomycota phylum as well. Another important pattern which we have observed is the presence of six amino acids in between the acid-base catalyst pair in all UPOs (Fig. 4) except some CPO sequences (*Clohesyomyces aquaticus, Rhizoctonia solani 123E, Rhizoctonia solani AG-3 Rhs1AP, Microdochium bolleyi, Penicillium camemberti, and Penicillium expansum*) placed among the UPOs, which seem to have seven amino acids (see Additional file 2: Figure S1). Besides, some of the new motifs (see Additional file 4: Figure S2) have also been found in different species of UPOs and formed a basis for their classification into five different subfamilies and a superfamily which encompasses both peroxygenase (*Mro*UPO) and peroxidase (*Lfu*CPO and other CPO sequences) named as Peroxidase-peroxygenase (Pog) superfamily.

### Classification of UPOs and CPOs

**Fig. 5:**
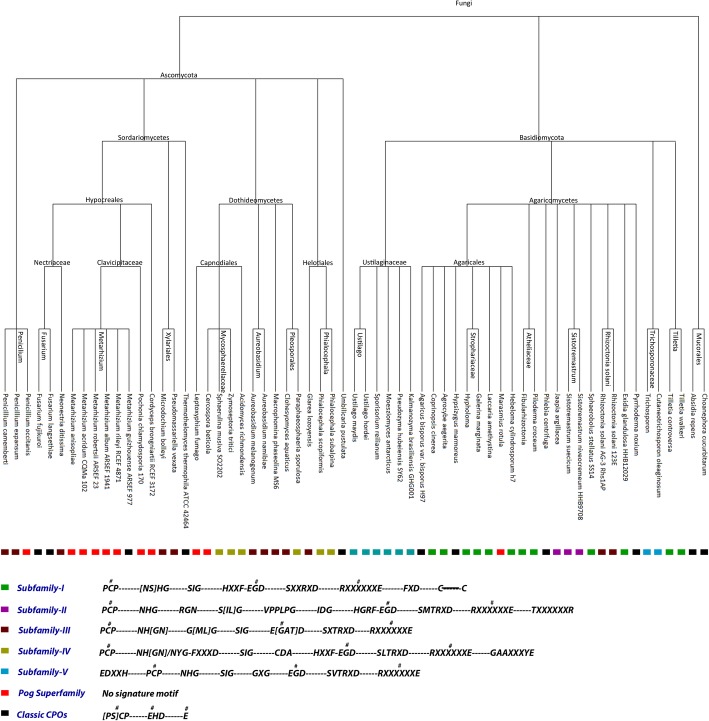
Species tree consisting of all the fungal species including all UPOs and CPOs belonging to different subfamilies and superfamily along with the motif patterns exhibited by each family. # signifies the motifs reported earlier in UPOs and CPOs

According to the phylogeny analysis, the CPOs which are considered as outgroup are classified into two families: classic CPOs and Pog superfamily, additionally, on the basis of newly found motifs, we classify UPOs into five different subfamilies (Fig. 5). Apart from the earlier recognized motifs, each UPO subfamily consists of its own signature motifs except the Pog superfamily which consists of the two experimentally validated species, namely, *Lfu*CPO and *Mro*UPO both of which have shown peroxygenating (comparatively lesser than that of *Aae*UPO) and peroxidase activities. *Lfu*CPO has been reported to show epoxidation and carbon-carbon bond cleavage [25] but with lesser potential than that of the *Aae*UPO [10]. The rest of the CPO sequences present in this family have not been explored yet in terms of their activities, therefore, we predict that these sequences in this superfamily may also be capable of showing peroxygenase and peroxidase activities as the *Lfu*CPO and *Mro*UPO. However, a detailed study is required to assess their properties and catalytic activities. The classic CPOs appear far from the UPOs showing the evolutionary distance among them. This family consists of the CPO motifs as reported previously [7].

### Selective pressure analyses

All branches were tested for the selection pressure in UPOs as well as in CPOs. According to the BUSTED method [26], gene-wide episodic diversifying selection in the branches at a *p*-value threshold of ≤0.05 was experienced (see Additional files 5 and 6). The site-specific method identified many functionally relevant sites which have experienced positive diversifying and negative purifying selection in UPOs and CPOs.

### Diversifying and purifying selection in UPOs

**Fig. 6:**
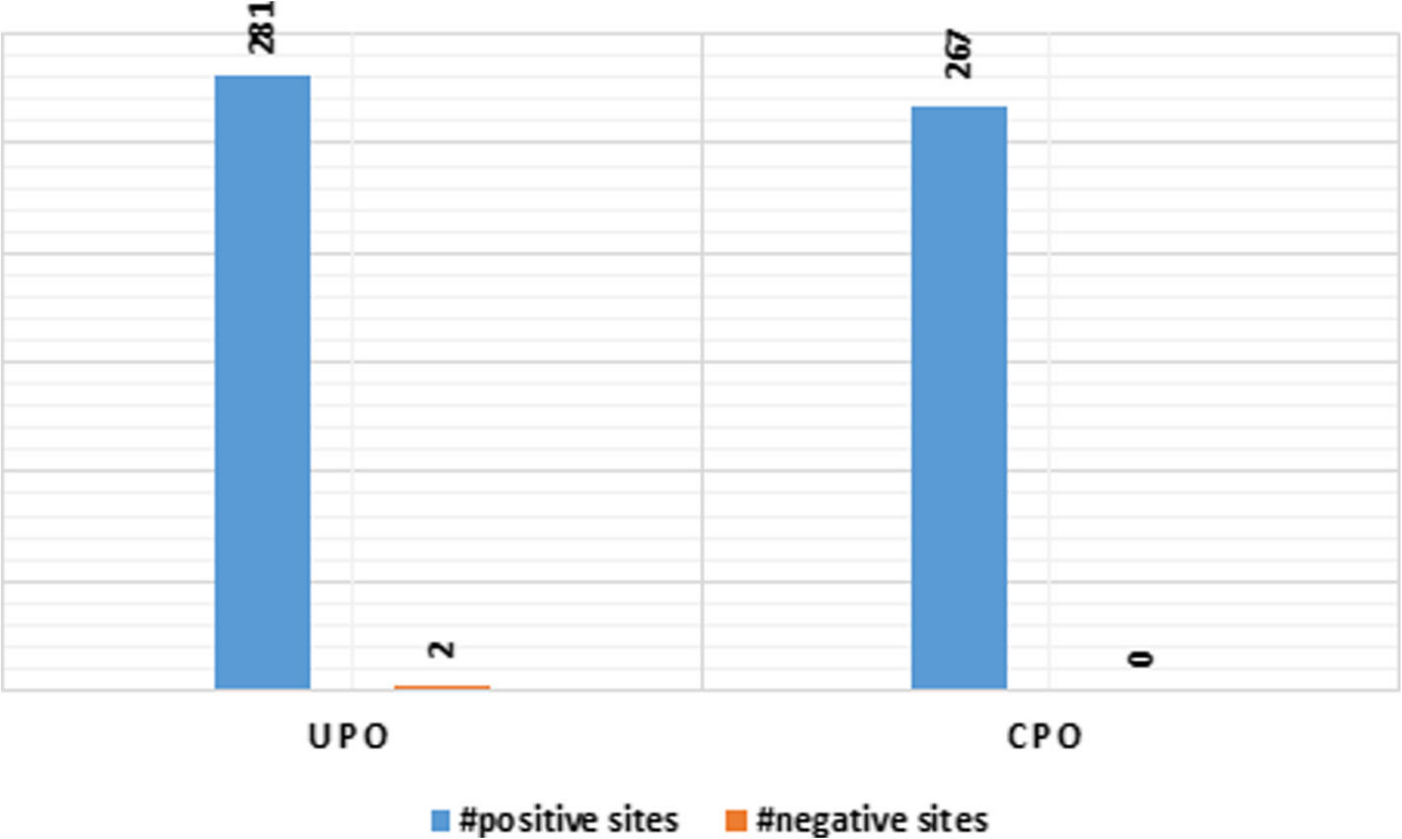
Bar chart showing a number of sites under positive and negative neutral selection in UPOs and CPOs

The strong purifying and diversifying selection has been detected on UPOs using different models (Additional file 7: Figure S3). Episodic diversifying/positive selection in UPOs at 248 different sites by MEME [27] at a p-value threshold of 0.05 and pervasive diversifying and purifying/negative selection has been identified by FUBAR [28] at 281 and 2 sites respectively at a posterior probability of 0.9 and above (see Additional file 5) (Fig. 6). According to site-specific method (MEME) results, UPOs exhibited synonymous substitutions higher than the nonsynonymous substitutions (see Additional file 8: Figure S4) and 30 functionally relevant sites with episodic and/ pervasive purifying and diversifying selection pressure were recognized from literature discussed as follows: Sites-90, 91, 93, and 100 lie in very close proximity of the PCP motif. Sites-143-145, 147, 149, and 151 showed positive selection which lies in close proximity to residues involved in interacting with the acetate substrate by making Van der Walls interactions between the methyl group and carboxyl C-atom of acetate and pyrrole ring of the heme system in *Aae*UPO [14]. Sites-184 and 185 in the alignment represent *Pro108* and *Pro109* undergone positive selection in UPOs which makes a *cis*-peptide bond with each other in *Aae*UPO. Also, the sites-280 to 283 and 285 showed diversifying selection. These sites represent the amino acids present between the acid-base catalyst pair in UPOs. Site-200 represents a well-known *Gly* residue in the EGD motif in UPOs, which has experienced a significant episodic diversifying selection. Sites-284 and 287 showed diversifying selection and site-289 showed purifying selection, all of them are involved in interacting with the aromatic rings in polycyclic aromatic hydrocarbons [14]. Sites- 396 and 445 represent two *Cys* residues involved in making a disulfide bridge to stabilize the C-terminal region in *Aae*UPO [14].

Some of the newly found motifs in the UPOs have also appeared to experience episodic and pervasive selection at different sites. Sites- 74-77 representing the first four residues in EDXXH motif of Subfamily- V UPOs have experienced episodic and pervasive diversifying selection. Site-107 which represents the *Gly* residue of [SN] HG in Subfamily-I of UPOs, NHG in Subfamily-II, III, and V, and NH [GN]/NYG in Subfamily-IV UPOs. Site- 118, 119, and 120 which represent the first three residues in the FXXXDG motif present in the Subfamily-IV of UPOs showed episodic and pervasive diversifying selection. Sites- 138 and 140 represents *Gly* residues in the G [ML] G motif in Subfamily-III of UPOs having experienced episodic and pervasive diversifying selection. Sites- 169 and 171 represent *Cys* and *Ala* residue in CDA motif present in Subfamily-IV of UPOs and showed episodic and pervasive diversifying selection. Sites-175 to 179 representing the last five residues in VPPLPG motif of Subfamily-II of UPOs showed pervasive and episodic diversifying selection. Similarly, sites- 182 to 184 which represent IDG motif of the same subfamily, have shown diversifying selection. Sites- 188 and 89 appear to have experienced episodic diversifying selection which represent the first and second *Gly* residue of GXG motif in Subfamily-V UPOs. Site- 197 represents the third residue in HXXF motif of Subfamily-I, II, and IV of UPOs. Sites- 204 and 207 representing amino acid residues present in the SXXRXD motif present in all UPOs showed diversifying selection. Sites- 285, 287–292 showed episodic and pervasive diversifying selection, where site- 289 showed pervasive purifying selection, represent the amino acid residues of GAAXXXYE motif present in Subfamily-IV of UPOs. Site- 295 represents the middle residue of FXD motif in Subfamily-I of UPOs. Sites- 335 to 338 and 341 represent amino acid residues in TXXXXXXR motif of Subfamily-II of UPOs showed episodic and pervasive diversifying selection.

The diversifying selection is seen on a large number of sites in the UPO alignment which indicates the spreading of more beneficial alleles throughout the sequences and also purifying selection which removes deleterious mutations [29].

The branch-site method (aBSREL) [30] found an evidence of episodic diversifying selection on 35 out of 36 tested branches in the phylogeny of UPOs at a *p*-value threshold of ≤0.05. The model fitted to the data with log likelihood are shown in Additional file 5 and the branches showing positive selection are shown in Additional file 9: Figure S5. The positive diversifying selection was detected on all tested branches except the three: *Aureobasidium melanogenum cbs110374* which belongs to Pezizomycotina subphylum of Ascomycota, *CPO Rhizoctonia solani 123E,* and *CPO Rhizoctonia solani AG-3 Rhs1AP.*

### Diversifying and purifying selection in CPOs including *Mro*UPO

In the case of CPOs, episodic diversifying selection has been identified at 166 different sites by MEME at a p-value threshold of 0.05 and pervasive diversifying and purifying selection has been identified by FUBAR at 267 and 2 sites respectively at a posterior probability of 0.9 and above (see Additional file 6 & Additional file 10: Figure S6) (Fig. 6). Similar to UPOs, CPOs also showed large number of synonymous substitutions greater than the nonsynonymous substitutions (see Additional file 11: Figure S7). We found 29 functionally relevant sites under positive selection, discussed as follows: Sites-95and 99 to 103 showed episodic and pervasive diversifying selection which are present right before and after the PCP motif respectively and are involved in providing rigid scaffolding for iron-sulfur interactions in *Lfu*CPO [31], and encompasses conserved *Cys* residue present in all HTPs and stabilizes the C-terminal region [7]. Sites-80 and 369 showing diversifying selection function as N-glycosylation sites in *Lfu*CPO. Site-96 represents the *Pro* in the PCP motif in CPOs which appeared to have experienced the episodic diversifying selection. Sites- 185 and 193 represent two *Cys* residues which form a disulfide bond in *Lfu*CPO. A *Ser* residue at site-216 showing episodic diversification forms a loop with the *Glu* residue of EHD motif in CPOs providing a primary set of interaction. Sites-206-208, 213, and 216 along with site-433 showed diversifying selection which function in forming a small channel connecting the heme distal site in CPOs [31]. Sites-209, 311, and 383 are identified with diversifying selection and these sites make interactions with organic substrates such as dimethylalanine which undergoes CPO-catalyzed oxidative demethylation [32]. Sites-392, 393, 395, 396, 403 to 405, 439, and 447 showed diversifying selection and are involved in binding with carbohydrates [31].

The branch-site method, aBSREL found evidence of episodic diversifying selection on 26 branches out of 31 branches tested as foreground in the phylogeny of CPOs at a *p*-value threshold of ≤0.05 (see Additional file 6). The model fitted to the CPO data with log likelihood are shown in Additional file 5. The branches showing positive selection are shown in Additional file 12: Figure S8. The branches with selection evidence include *Marasmius rotula, Metarhizium guizhouense ARSEF 977, Metarhizium album ARSEF 1941, Penicillium occitanis, Pochonia chlamydosporia 170, Phellinus noxius, Penicillium expansum, Leptoxyphium fumago, Umbilicaria pustulata, Thermothelomyces thermophila ATCC 42464, Pseudomassariella vexata, Clohesyomyces aquaticus, Macrophomina phaseolina MS6, Phlebia centrifuga, Choanephora cucurbitarum, Metarhizium rileyi RCEF 4871, Microdochium bolleyi, Metarhizium anisopliae, Cordyceps brongniartii RCEF3172, Fusarium langsethiae, Cercospora beticola, Absidia repens, Metarhizium acridum CQMa 102, Hypsizygus marmoreus, Penicillium camemberti,* and, *Fusarium fujikuroi.* The negatively selected branches include *Metarhizium robertsii ARSEF 23, Agaricus bisporus,* and *Agaricus bisporus var. bisporus H97.*

### Functional divergence analyses

**Fig. 7:**
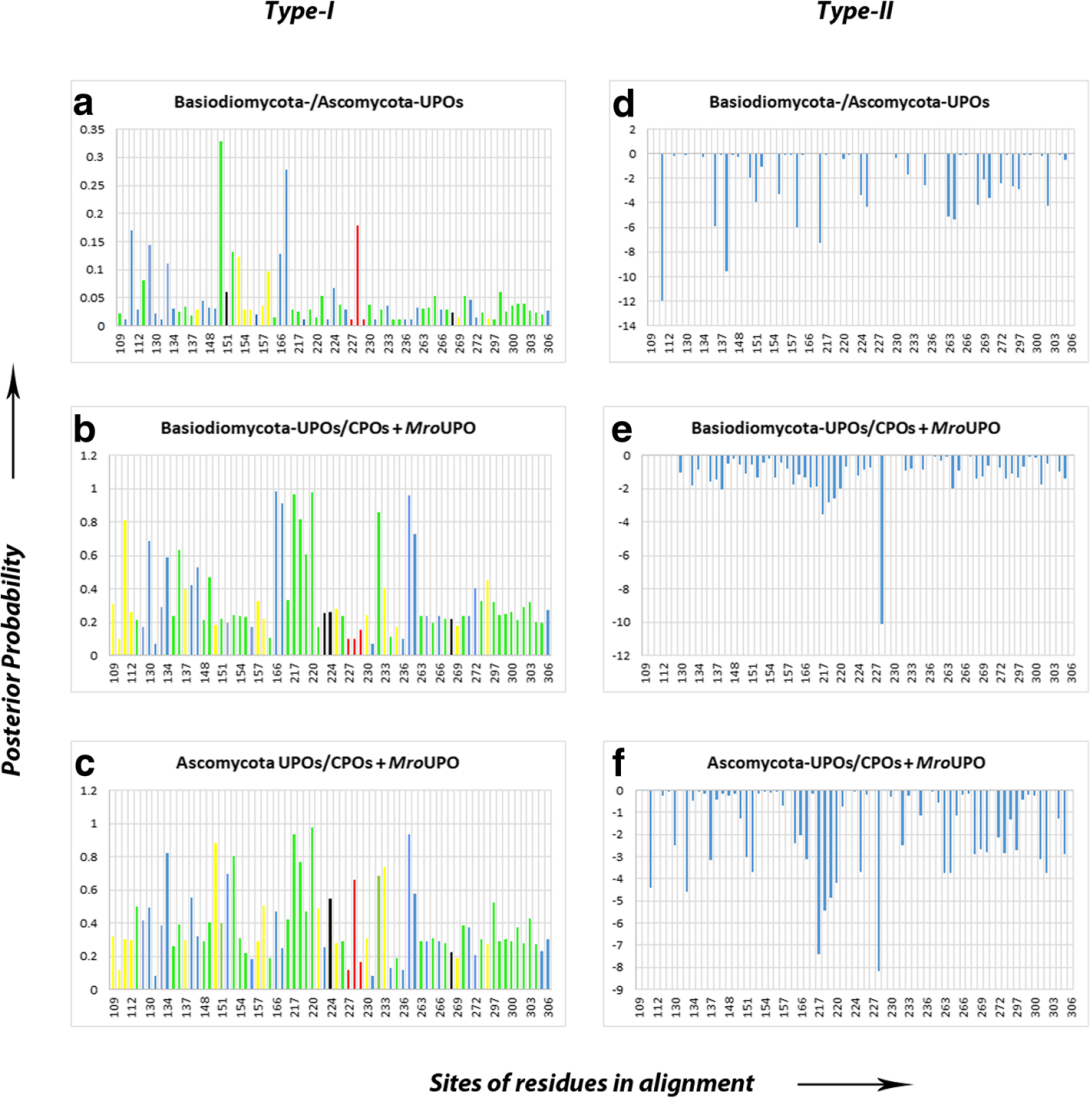
Column graphs showing Type-I (**a**-**c**) and Type-II (**d**-**f**) functional divergence analysis on CPOs and UPOs. *Red* bars represent the sites having EGD motif in UPOs and EHD motif in CPOs, the *black* bar represents the significant and functionally relevant sites, *green* bars indicate the positively selected sites showing functional divergence, *yellow* bars indicate the sites showing positive selection along with functional divergence, and *magenta* colored bars indicate sites showing negative selection

**Fig. 8:**
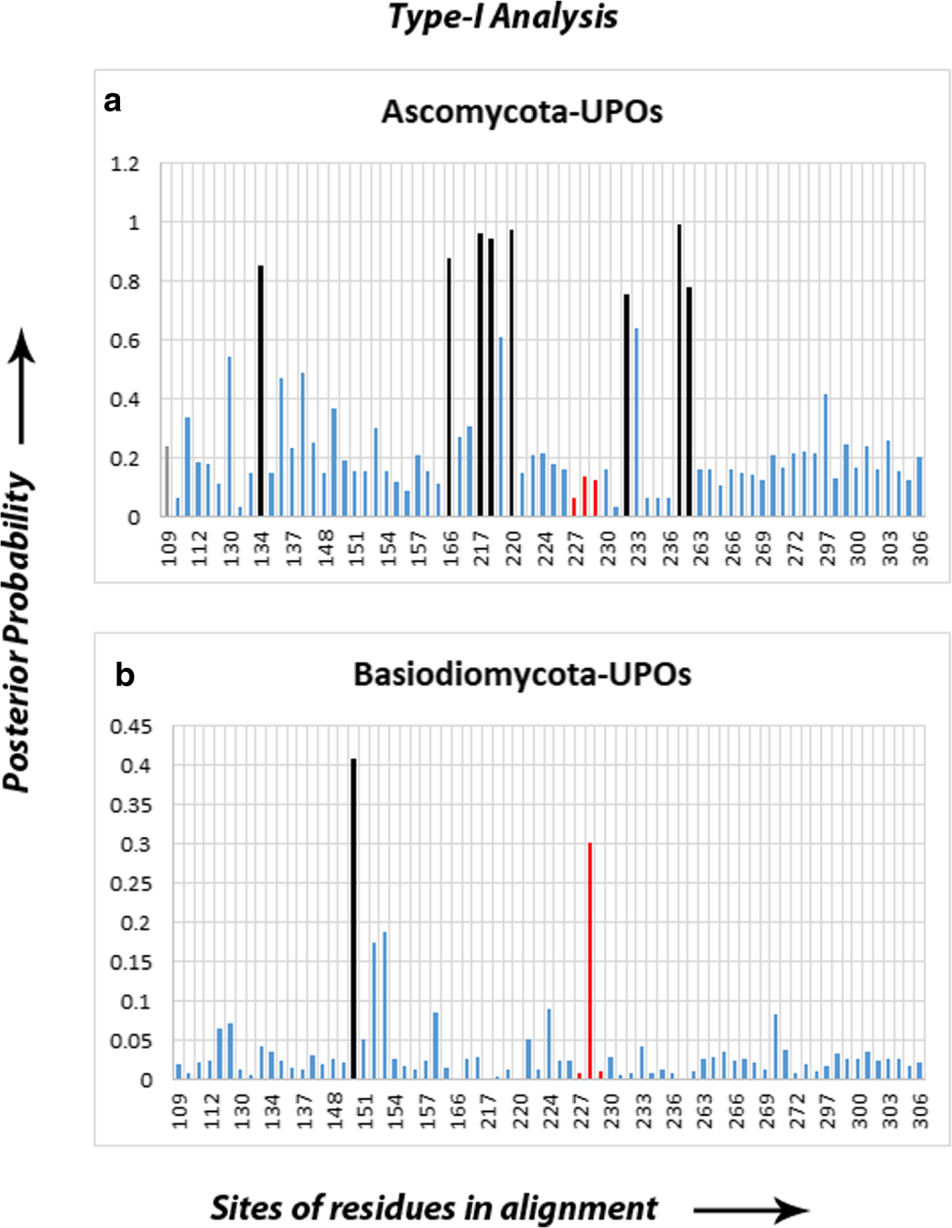
Column graphs showing the critical amino acids involved in functional divergence in (**a**) Ascomycota UPOs and (**b**) Basidiomycota UPOs. *Red* colored bars indicate the EGD motif in UPOs and *black* bars represent the newly found critical sites which are involved in functional divergence

Type-I functional divergence (also known as site-specific rate shift) was observed in all the pairs but no significant radical (Type-II) divergence was observed (Table 1). The sites in all clusters show the typical pattern of Type-I functional divergence which signifies the distribution of diverse amino acid residues in one cluster (tested cluster) and conserved amino acid residues in another cluster (background cluster) (see Additional file 13: Figure S9). 13 functionally relevant sites have been identified with a significant posterior probability in UPOs as well as in CPOs showing the Type-I functional divergence (Fig. 7). The Type-I analysis on the clusters revealed that the CPOs are more conserved than the UPOs whereas the Basidiomycota UPOs and Ascomycota UPOs showed functional divergence with respect to CPOs along with *Mro*UPO (Fig. 8).

**Table 1:** Showing the Ө_I_ and Ө_II_ values of all the pairs studied

Species	Ө_I_ ± Ө_SE_	Ө_II_ ± Ө_SE_	#Sites with no change	#Sites with radical change	#sites with conservative change
Basidiomycota- vs Ascomycota- UPOs	0.048048 ± 0.073911	−1.788713 ± 0.819243	39.411765	18.705882	14.882353
Basidiomycota UPOs vs CPOs	0.342599 ± 0.062568	−0.902184 ± 1.326494	27.352941	27.705882	17.941176
Ascomycota UPOs vs CPOs	0.387130 ± 0.084127	−1.640309 ± 1.841228	26.764706	27.941176	18.294118

There was no significant type-II functional divergence

### Type-I functional divergence (site-specific rate shift) in UPOs

UPOs were observed to exhibit shifted functional constraints at the following sites: Sites-109 to 112 lies close to the PCP motif, site-150 and 155 are present close to the residues involved in making van der Waal’s interaction with acetate substrate in UPOs. Sites- 148-150 which signify the newly found motif G[ML]G in the Subfamily-IV UPOs, showed significant functional divergence with the posterior probability of 0.32, 0.47, and 0.88 respectively. Sites- 166 and 167 show functional divergence with the posterior probability above 0.9 in the Basidiomycota UPOs, where these sites represent the *Gly* and *Asn* in the RGN motif in Subfamily-IV of UPOs. Sites- 216-218 signify GXG motif in Subfamily-V of UPOs and showed significant values for site-specific rate shift in Basidiomycota and Ascomycota UPOs. The SXXRXD motif found in all UPOs also showed significant functional divergence at all of its residues except the first *Ser.* The sites-226, 234, 235, and 236 lies in close proximity to the EGD motif. The *Gly* residue in EGD motif in Ascomycota UPOs showed a significant functional divergence with a posterior probability of 0.66 which is involved in site-specific rate shift UPOs from the CPOs.

### Type-I functional divergence (site-specific rate shift) in CPOs

Ascomycota UPOs vs CPOs including *Mro*UPO pair shows a Type-I functional divergence at several functionally relevant sites such as sites-217-221 and 223–226 which lie close to the EHD motif in CPOs (including *Mro*UPO) with a posterior probability ranging between 0.5–09. Most importantly, this pair showed a significant functional divergence at the site-228, which represents the *His* and *Gly* residues in CPOs and Ascomycota UPOs respectively with a posterior probability of 0.6. Another functionally relevant site includes site-227 which makes a hydrogen bond with *Gln* located at a distance and maintains its orientation and provides charge-charge interactions.

### Type-I analysis

The Type-I Analysis shows site-specific profile predicting critical amino acid residues responsible for site-specific rate shift in the enzymes (see Additional file 14). According to the results, the CPOs cluster has not shown any divergence but the other two clusters, i.e., Basidiomycota UPOs and Ascomycota UPOs have shown some specific sites with significant posterior probabilities (Fig. 8).

### Ascomycota UPOs

The Ascomycota UPOs showed 8 specific residues which have experienced functional divergence with respect to the CPOs and may have been functionally relevant in their catalytic roles. Site-134 shows a consistent pattern of *Ile/Val/Leu* in the CPOs but showed substitutions in the Ascomycota UPOs replaced by *Val/Thr/Ala/Ser/Gly.* Similarly, another site-166 shows the presence of only two residues, namely, *Gly/Ala* in Basidiomycota UPOs and no particular residues in Ascomycota UPOs and CPOs. Site-217 shows the conserved *Leu* in CPOs but no particular distribution of residues in Ascomycota UPOs. Site-218 shows the presence of *Gly* in Ascomycota UPOs as well as in Basidiomycota UPOs but no specific residues in CPOs. Similarly, site-219 shows *Leu/Ile* residues in Ascomycota UPOs and Basidiomycota UPOs but any such specific residues are not present in CPOs at this site. Site-232 does not show a particular residue distribution but it lies in close proximity to EGD and/or EHD motif in UPOs and CPOs respectively. Site-261 represents the conserved *Gly* residue in Ascomycota as well as in Basidiomycota UPOs but no specific residues in CPOs were found distributed except in the *Mro*UPO which also consists of *Gly* at the same site. Site-262 shows the distribution of *Asn/Asp* in Basidiomycota UPOs, *Asn/Asp/Gln* in Ascomycota UPOs, and no specific pattern in CPOs.

### Basidiomycota UPOs

Basidiomycota UPOs have shown 2 specific residues which are involved in functional divergence. One of these sites, site-228 represents the *Gly* residue in the UPOs and *His* residue in CPOs and *Mro*UPO. Another site-150 shows the presence of *Gly/Ala* in Ascomycota UPOs but not any specific distribution of residues in CPOs were found at the same site.

## Discussion

### Distribution and functional evolution of fungal UPOs

Fungal species exhibiting UPO identified in the subphyla of Basidiomycota and a single subphylum of Ascomycota appear very diverse in their characteristics, habitats, and behaviors. Among the obtained UPO sequences, three species belonging to Pezizomycotina are plant pathogenic: *Neonectria ditissima* causes cankers in Apples [33], *Zymoseptoria tritici* causes leaf blotch in wheat [34], and *Sphaerulina musiva* causes leaf spot and canker disease in poplar trees [35]. Besides, UPO sequences were also found in some extreme environment surviving species such as *Aureobasidium melanogenum cbs110374*, *A. namibiae cbs147.97,* which can tolerate up to 10% NaCl and grows between 10 and 35 degree Celsius [36], *Acidomyces richmondensis* which is adapted to extremely acidic (pH < 1) and thermophilic (40–50%) environment of acid mine drainage [37], and *Phialocephala subalpina,* which is a dark septate endophyte that increases plant tolerance against salt or drought [38]. The species belonging to Ustilaginomycotina are mostly plant parasitic in nature. *Tilletia walkeri* also known as Ryegrass bunt and *T. controversa* both are plant pathogens. Other pathogenic species belonging to Ustilaginomycotina include *Ustilago maydis*, *U. hordei* which cause corn smut on maize and barley respectively [39]*,* and *Sporisorium reilianum* which infects maize and sorghum leading to head smut in both [40]. Another pathogenic species found to have UPO is *Mixia osmundae iam14324* which is an intracellular parasite of ferns, belongs to Pucciniomycotina. Interestingly, none of the fungal species consisting of UPO obtained in this study is edible. *Aureobasidium melanogenum cbs110374* also known as *Aureobasidium pullulans var melanogenum cbs110374* is also responsible for causing infections in humans [36]. Besides, the *A. pullulans* infection potential is partially linked to the production of some extracellular enzymes [41]. However, *Glarea lozoyensis atcc20868* has medical relevance due to its ability to produce natural antifungals named pneumocandins, which act by inhibiting fungal β-(1,3)-glucan synthesis [42] and has provided antifungal therapy to treat life-threatening fungal infections [43]. Another non-pathogenic fungal species having UPO is *Pseudozyma hubeiensis sy62* which produces a large number of extracellular glycolipids, saccharides, and mannosylerythritol lipids from vegetable oils. The different characteristics and behavior of the fungal species having UPOs show their wide and diverse distribution in the fungal kingdom.

### Hypothesized functional roles of newly found conserved motifs in UPOs

As implicated by earlier publications, the UPOs have three conserved motifs: PCP, EGD, and R-E. Here, we have found some new conserved motifs among different subfamilies of UPOs and mapped them on to the experimentally resolved structure of *Aae*UPO including the other modeled structures of newly found UPOs (Additional file 15: Figure S10) revealing close proximity to the binding site in the enzymes. Therefore, we postulate that they may play an important role in substrate binding and specificity. These motifs are discussed as follow: NHG/NHN/SHG motif may be responsible for actively participating as the active and binding residues as *Asn, His,* and *Gly* are supposed to be involved as the binding residues in proteins, and *Ser* is capable of forming H-bonds with polar substrates. The S [IL] G motif lying in between the PCP and the EGD motifs. This SIG motif lies in close proximity to the binding pocket of the enzyme. We predict that the role of this motif might be related to its specificity for the substrates as *Ser* is slightly polar in nature capable of forming hydrogen bonds with various polar substrates, and *Gly* contains a hydrogen as its side chain which could provide conformational flexibility, and *Ile* is an aliphatic hydrophobic amino acid which could be involved in the binding and recognition of hydrophobic ligands such as lipids [44]. However, the above mentioned three species consists of *Leu* instead of *Ile*, which is also hydrophobic in nature and may be involved in as the same function as the *Ile*. The SXXRXD motif in all UPOs may be responsible in providing stability to the structure and forming H-bonds with a variety of polar substrates as *Ser, Thr,* and *Asp* are involved in forming H-bonds with the polar substrates and to provide protein stability. Another conserved sequence motif which was recognized through sequence analysis of all UPOs was the presence of six amino acids in between the acid-base catalyst pair. The occurrence of the PCP and EGD motifs is same in all fungal species consisting of UPO but the species from different subphyla follow a different pattern of amino acid residues arrangement present in between the acid-base catalyst pair except for two species *Tilletia walkeri* and *Tilletia controversa* from Ustilaginomycotina*,* which follows more similar pattern as the Agaricomycotina UPO consisting species than those belonging to Ustilaginomycotina of Basidiomycota (see Additional file 2: Figure S1). However, there is no consistent presence of a specific pattern of amino acid residues in between the acid-base pair. The hypothesized functions of the UPOs belonging to different subfamilies are summarized in Table 2 based on the roles of amino acid residues present in the motifs.

**Table 2:** Hypothesized functions of the classified subfamilies of UPOs based on the roles of amino acid residues present in the motifs

Subfamily	Motif	^a^Roles of amino acids present in the motif	Hypothesized functions of the subfamily
I	FXD	*Phe* is basically involved in stacking interactions with other aromatic side-chains and the *Asp* is frequently involved in salt-bridges interacting with positively charged amino acids to create stabilizing H-bonds which can be important for protein’s stability.	may actively involve in interacting with aromatic residues and in forming stable H-bonds imparting to the structural stability.
	*Cys-Cys*	disulfide bond is mostly involved in providing stability to protein structure.	
II	RGN	*Arg* is frequently involved in making salt-bridges with the negatively charged amino acids creating stable H-bonds which may be crucial for the structure stability; the *Gly* provides the conformational stability, and the *Asn* is involved as protein’s active and binding sites.	may potentially interact with the hydrophobic ligands such as lipids and may show specificity for some polar substrates.
	IDG	*Ile* in the IDG motif is involved in recognizing hydrophobic ligands; *Asp* forms stable H-bonds with positively charged amino acids required for protein’s stability, and the *Gly* again may provide conformational stability.	
	TXXXXXXR	*Thr* is often found in protein centers and capable of forming H-bonds with the polar substrates.	
III	G [ML]G	the *Gly* provides the conformational stability, *Met* and *Leu* plays a role in binding and recognition of hydrophobic ligands.	may play important role in substrate specificity/recognition and capable of forming strong H-bonds with the polar substrates.
IV	CDA, FXXXDG, GAAXXXYE, and HXXF	*Ala* is involved in substrate recognition and specificity; *Tyr* makes stacking interactions with the aromatic side chains; *His* is involved in protein metal binding sites; and *Phe* also makes stacking interactions with aromatic side chains.	may show large interactions with the aromatic substrates and these motifs are perhaps involved in substrate recognition and binding.
V	EDXXH	*His* is most commonly involved in active and binding sites especially in metal binding sites and the *Asp* and *Glu* residues create the stable H-bonds.	may play an important role in reacting with positively charged amino acids.
	GXG	*Gly* provides the conformational stability	

^a^refers to [44]

### Phylogenetic inference of UPOs

The phylogeny of UPOs suggests that most of the highly similar UPOs with respect to *Aae*UPO belong to Agaricomycotina followed by Ustilaginomycotina, and Pucciniomycotina subphyla of Basidiomycota and Pezizomycotina subphylum of Ascomycota. However, four of the resultant UPOs (*Glarea lozoyensis atcc20868, Aureobasidium melanogenum cbs110374, Aureobasidium namibiae cbs147.97,* and *Neonectria ditissima*) that belong to Ascomycota are placed in a cluster along with CPOs in the phylogenetic tree which indicates that they may show less *Aae*UPO-like activity than that of *Mro*UPO and *Lfu*CPO. Besides, the *Mro*UPO is placed in between the Ascomycota UPOs and the Ascomycota CPOs all of which belonging to Pezizomycotina subphylum. This clearly indicates the intermediate state of the *Mro*UPO existing between the CPOs and UPOs. However, these CPOs belong to the Pog superfamily which surpasses both peroxygenase and peroxidase suggesting that the other CPO sequences may also exhibit peroxygenase properties. Additionally, some of the CPO sequences are placed along with UPOs, which on further analysis, appeared more like a kind of UPOs.

Most of the resultant highly similar UPOs are “gilled mushrooms” which are most commonly found in nature. It is worth noting that only a single UPO species i.e., *Mixia osmundae iam14324* is present in Pucciniomycotina and most of the fungal species exhibiting UPOs are plant pathogenic and/or harmful in nature. The most highly similar fungal species to *Aae*UPO, i.e., *Galerina marginata cbs339.88* is extremely poisonous species from the Hymenogastraceae family of the order Agaricales. Although *Aae*UPO is not plant parasitic fungus, the majority of the other obtained UPOs are pathogenic in nature which may indicate an early evolutionary origin of UPOs. It also suggests that UPO is widespread in Basidiomycota of fungal kingdom exhibiting different kinds of species showing variant behavior and adaptations. Besides, the *Agaricus* genus found consists of CPO and UPO in different strains which may be linked to their adaptable behavior.

### Diversifying and purifying selection among UPOs and CPOs

The UPOs and CPOs are very distant in terms of their divergence which may result in the saturation of dS. Therefore, to avoid this, we analyzed a large set of sequences of UPOs (44 sequences) and CPOs (23 sequences) that helped in providing more reliable information. Secondly, terminal branches were selected for dS and dN estimation avoiding the interspecific branch lengths. Besides, the higher dN/dS ratio detected at a relatively lower level of divergence (dS and dN < 0.1) provides a strong evidence that the higher inferred intensity of selection in UPOs and CPOs was not caused by the saturation of dS.

A strong diversifying and purifying selection was observed at various functionally relevant sites in UPOs and CPOs. These sites include *Gly* in the NHG/SHG/NHN motif in all UPOs, EDXX in the EDXXH motif found only in Subfamily-V UPOs, *Gly* in the G[ML]G motif found in Subfamily-III UPOs, IDG motif of Subfamily-II UPOs, FXX and *Cys* and *Ala* in the FXXXDG and CDA motif in Subfamily-IV UPOs respectively, the *Gly* residue in the well-known EGD motif in all UPOs, and residues present between the acid-base catalyst. These motif sites have experienced strong diversifying selection indicating the spread of beneficial alleles and given the supposition of their roles in substrate specificity, recognition, and structure stability, they may have also stabilized the function of catalyzing a specific substrate among the UPOs. On the other hand, in CPOs, several sites which are relevant in stabilizing the structure and substrate binding have experienced diversifying selection. We postulate that these positively selected sites in UPOs and CPOs have significantly contributed to the evolution of their functional diversity.

### Functional divergence among UPOs and CPOs

The proportion of fixed radical change (F00,R) and conserved change (F00,C) was zero in all pairs indicating a radical functional conservation in UPOs and CPOs. The Basidiomycota UPOs cluster showed site-specific rate shift with respect to CPOs and *Mro*UPO cluster*,* and same was followed by the Ascomycota UPOs showing even higher values of functional divergence including the EGD and EHD motifs in UPOs and CPOs respectively. The former cluster showed a very small value (0.09) of Type-I functional divergence on *His/Gly* residues in EHD/EGD motifs as compared to the latter (0.62) indicating Basidiomycota UPOs as more conserved as compared to the Ascomycota UPOs. According to the Type-I Analysis, CPOs cluster has not shown any functional divergence but was shown by Basidiomycota UPOs and Ascomycota UPOs with respect to CPOs including *Mro*UPO, which confirms the divergence of UPOs from CPOs without any rapid evolution. Besides, some sites were predicted with higher values of posterior probabilities other than the EHD motif in CPOs, which indicates that there are relevant sites other than the EHD/EGD motif which might be responsible for different catalytic activities of CPOs and UPOs (Fig. 8).

### Intermediate behavior of *Mro*UPO ranging between UPO and CPO

Among the predicted critical amino acid residues in Ascomycota UPOs and Basidiomycota UPOs (posterior probability ranging between 0.8–0.9), most of the residues in *Mro*UPO followed as the same pattern as the CPOs. For example, site-134 in CPOs showed *Ile/Val/Ala/Gly* and *Mro*UPO exhibits an *Ile* at this site while none of the UPOs from Basidiomycota or Ascomycota exhibits an *Ile*. At another site-217, both CPOs and *Mro*UPO consist a *Leu* but no specific pattern in UPOs. Site-218 exhibits a conserved *Gly* in UPOs but no such residue exists in CPOs and *Mro*UPO. Similarly, site-219 consists of *Leu/Ile* in UPOs but not in CPOs and *Mro*UPO. Only one such site in *Mro*UPO resembled the pattern of UPOs at the site-261 identified at a posterior probability of 0.99, which also consists of a conserved *Gly* in UPOs and in *Mro*UPO as well. The function of these sites is unknown but may be responsible for the distinct behavior of *Aae*UPO, *Lfu*CPO, and *Mro*UPO.

### Correlation between positive/diversifying selection and functional divergence

**Fig. 9:**
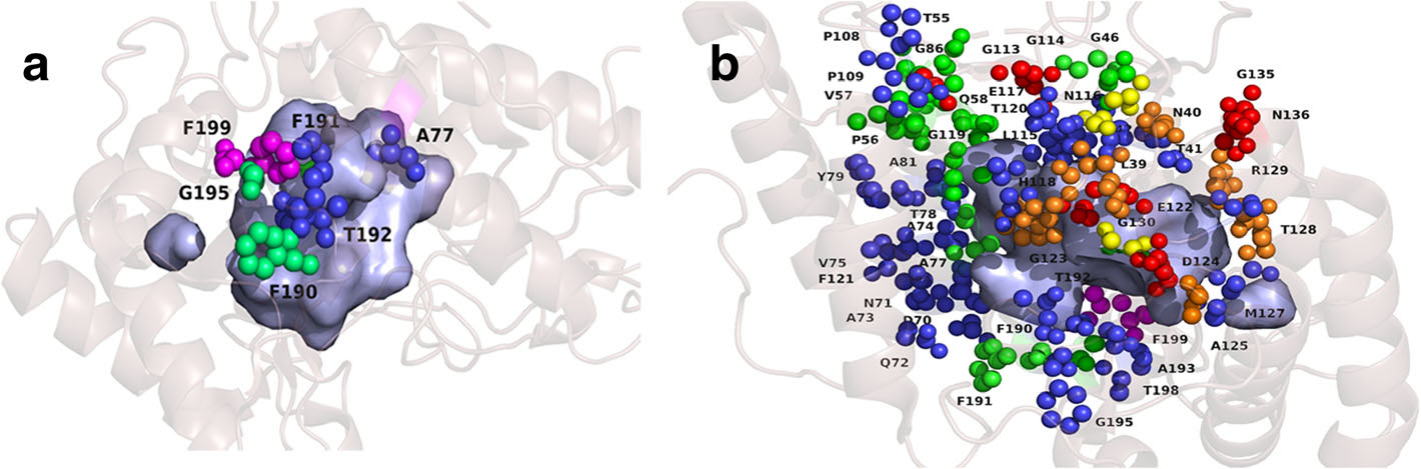
showing functionally divergent sites with selection pressure in *Aae*UPO **a**) showing the residues which interact with aromatic rings in polycyclic aromatic hydrocarbons, and **b**) showing other relevant sites. The surface area represents binding pocket and residue sites are represented as small spheres. *Orange* spheres show Type-I functional divergence sites, *green* spheres indicate positive selection, *blue* spheres indicate the sites with positive selection and Type-I functional divergence, *red* spheres represent the predicted specific residues responsible for functional divergence in UPOs, *yellow* spheres indicate residues with positive selection+functional divergence+predicted critical residue and *magenta* colored spheres indicate negative selection

The positive and negative selection sites were also mapped at the sites showing functional divergence in UPOs and CPOs (Fig. 7). Our results show that there are a large number of sites under selection pressure as well as the functional divergence together. These sites including other predicted critical residues in UPOs were later mapped on to the three-dimensional structure of *Aae*UPO (Fig. 9). Some of these sites interact with the aromatic rings in polycyclic aromatic hydrocarbons [14] and the function of rest of the other sites is unknown. Interestingly, one of these functionally unknown sites, *Thr55* in *Aae*UPO showed positive selection and functional divergence and was as well predicted to be a critical amino acid residue possibly responsible for driving the functional divergence of UPOs from the CPOs. The sites showing selection pressure and functional divergence are majorly distributed around the binding region of *Aae*UPO and hence, we postulate that they might be involved in making interactions with the substrates. As positive selection leads to the spreading of advantageous mutations, it has been found associated with the protein functional shifts [45]. As evident from the results, Ascomycota and Basidiomycota UPOs clusters showed diverse amino acids in opposition to CPOs cluster, also showing diversifying selection may have resulted into the functional divergence among the UPOs.

## Conclusion

In this study, we have found 113 putative fungal UPOs from 35 different species (including strains) distributed in Basidiomycota and Ascomycota phyla. The single sequences from each species were thoroughly analyzed and studied for their phylogeny along with CPOs and further, analyzed for selective pressure and functional divergence. Here, we report seven novel findings: (1) 113 new putative fungal UPO encoding sequences, (2) five subfamilies of UPOs and a superfamily on the basis of phylogeny and the motif patterns, (3) 16 new conserved motifs present in all and/or subfamilies of UPOs which are hypothesized to be involved in substrate specificity, recognition, and binding, (4) existence of a specific pattern having six amino acid residues in between the acid-base catalyst pair in UPOs, (5) pervasive diversifying and purifying selection among UPOs and CPOs, (6) site-specific functional shift in CPOs and UPOs suggesting their evolution from the CPOs and *Mro*UPO being an intermediary state between the two, and (7) some predicted critical amino acid residues in UPOs other than the known motifs, which could have been responsible for their functional diversity.

However, the main physiological role of UPOs in fungi remains unknown but according to the *Aae*UPO catalyzed reactions could be bio-physiologically related to some special habitats with high amounts of aromatic compounds and lignocellulose fragments which can be utilized as a carbon source. So far studied UPOs exhibit a vast variety of properties such as *Aae*UPO and *Mro*UPO. Similarly, newly obtained fungal UPOs may exhibit far more interesting properties. They have a wide range of possibilities in synthetic chemistry such as efficient enantioselective oxidations of bulk and fine chemicals, development of personalized drugs and reference metabolites, and biomimetics. The future works involve the detailed study of gilled mushrooms consisting of UPOs, features of different subfamilies of UPOs, the cause of their similarities and/or differences among them, a comparative study with the other species which are found lacking the UPOs, and a detailed study on the Pog family. This study has provided more UPOs which can be studied further in details to identify the physiological role of UPOs in fungal species.

## Methods

### Overview of the analysis pipeline

Various *in-silico* filters were applied to mine all the genome sequences for obtaining relevant sequences similar to *Aae*UPO. The protein crystal structures of the obtained UPO encoding sequences were predicted to analyze their binding pockets. Further, these obtained sequences were subjected to phylogenetic analysis to study their relatedness with CPOs and distribution among the fungal phyla. The resultant phylogeny was analyzed for the signature of Long Branch Attraction (LBA) to avoid any false conclusion regarding the evolutionary relationships. Further, the sequences of obtained fungal UPO species were thoroughly analyzed to study the arrangement of motif patterns and differences among the UPOs belonging to different subphyla. The UPO and CPO sequences were analyzed for selection pressure to identify the positive diversifying or negative purifying selective sites and branches in phylogeny and were subjected to functional divergence study. Further, sites with selection pressure and functional divergence in UPOs were also mapped to the structure of *Aae*UPO as well as the multiple sequence alignment of all UPOs and CPOs.

### Data collection

Peptide sequences of all fungal genomes constituting 812 different species (or strains) were downloaded from the Ensembl fungal genome database via FTP (ftp://ftp.ensemblgenomes.org/pub/) [46]. The details of the fungal genomes are provided in Additional file 16: Table S3. The fungal CPO sequences representing a complete CPO protein were downloaded from NCBI [47], details are provided in Additional file 17: Table S4.

### Homology search and identification of UPO motifs

We created a pipeline for the identification of UPOs in fungal genomes. *Aae*UPO was used as a query to perform a similarity search using PHMMER (http://hmmer.org; version 3.1b2) against the generated fungal genomes database with an E-value of 10.0 and an inclusive E-value of 0.01 giving ~ 1 false positive in every 100 searches with different query sequences which were further filtered by sequence-based clustering using cd-hit software [48] at 90% similarity cut-off and a word-length of 5 residues providing representatives of the clustered families. The obtained sequences were further subjected to graph-based clustering using MCL software [49] at an inflation value of 1.4 to eliminate the dissimilar sequences corresponding to the seed sequence. The resultant sequences were then searched for motifs providing the final resultant UPO-encoding sequences. The most highly similar sequences among these obtained putative sequences were selected using ClustalX2 [50] by calculating the similarity of putative sequences belonging to a specific species with respect to the *Aae*UPO protein sequence.

### Structure prediction

The protein structures of the finally obtained 35 highly similar sequences were predicted using SwissModel [51] and their binding cavities were analyzed for the presence of surrounding aromatic residues as found in *Aae*UPO using Pymol [52]. The predicted model template and percent identity are provided in Table 3.

**Table 3:** Summarizes the details of resultant UPO sequences showing their molecular weights and sequence identity with the template used to predict their structures

Peptide sequence	Fungal species	Number of Amino acids	Molecular weight (kDa)	Kind of UPO (based on structure template)	Sequence Identity with the template (%)
PzperKZV89105	*Exidia glandulosa hhb12029*	357	38.5	*Aae*UPO	58.62
EKM83318	*Agaricus bisporus var burnettii jb137s8*	359	39.28	*Aae*UPO	65.74
GAC96564	*Pseudozyma hubeiensis sy62*	242	26.45	*Mro*UPO	27.52
KIM43689	*Hebeloma cylindrosporum h7*	366	39.65	*Aae*UPO	62.93
KZS95533	*Sistotremastrum niveocremeum hhb9708*	350	37.19	*Aae*UPO	41.81
KDQ50980	*Jaapia argillacea mucl33604*	323	35.14	*Aae*UPO	38.46
KIM85662	*Piloderma croceum f1598*	358	39.89	*Aae*UPO	54.11
KZP09816	*Fibulorhizoctonia sp cbs109695*	366	39.19	*Aae*UPO	55.76
EJT50684	*Trichosporon asahii var asahii cbs2479*	210	23.67	*Mro*UPO	35.23
KIS69141	*Ustilago maydis*	265	29.24	*Aae*UPO	35.00
CCF52713	*Ustilago hordei*	244	27.13	*Mro*UPO	26.15
KIJ40725	*Sphaerobolus stellatus ss14*	367	39.03	*Aae*UPO	66.56
EAU83038	*Coprinopsis cinerea okayama7.130*	349	38.15	*Aae*UPO	63.58
EKC98656	*Trichosporon asahii var asahii cbs8904*	213	23.51	*Mro*UPO	40.96
KDR72025	*Galerina marginata cbs339.88*	361	39.04	*Aae*UPO	71.52
KLT38585	*Cutaneotrichosporon oleaginosus*	216	24.06	*Mro*UPO	29.35
KJA15764	*Hypholoma sublaterium fd334ss4*	362	38.71	*Aae*UPO	63.84
CBQ73518	*Sporisorium reilianum*	241	26.46	*Mro*UPO	27.06
EST07231	*Kalmanozyma brasiliensis ghg001*	270	29.27	*Aae*UPO	36.54
EPE26857	*Glarea lozoyensis atcc20868*	271	29.42	*Aae*UPO	31.22
KEQ59606	*Aureobasidium melanogenum cbs110374*	227	24.25	*Aae*UPO	29.95
GAA97831	*Mixia osmundae iam14324*	189	20.26	*Aae*UPO	33.33
KIK06072	*Laccaria amethystine laam08.1*	366	39.61	*Aae*UPO	67.08
Mycgr3P74298	*Zymoseptoria tritici*	223	24.49	*Mro*UPO	32.16
OAG12657	*Paraphaeosphaeria sporulosa*	219	23.54	*Aae*UPO	36.92
KPM41744	*Neonectria ditissima*	269	28.77	*Aae*UPO	33.91
KUJ12795	*Phialocephala scopiformis*	208	22.54	*Aae*UPO	34.31
OAJ19358	*Tilletia walkeri*	360	38.19	*Aae*UPO	56.78
KEQ68786	*Aureobasidium namibiae cbs147.97*	214	22.53	*Aae*UPO	33.15
GAK67998	*Moesziomyces antarcticus*	245	26.74	*Aae*UPO	35.68
KXL42501	*Acidomyces richmondensis*	266	28.83	*Aae*UPO	33.61
OAJ12013	*Tilletia controversa*	359	38.48	*Aae*UPO	55.21
CZR67509	*Phialocephala subalpina*	208	22.72	*Aae*UPO	32.18
EMF09816	*Sphaerulina musiva so2202*	250	27.11	*Mro*UPO	31.92
KZT41007	*Sistotremastrum suecicum hhb10207ss3*	292	31.71	*Aae*UPO	38.43

### Phylogeny analysis

The phylogeny analysis was done using Mega7 software [53]. A best-fit model was selected using ProtTest [54] 3 which recommended LG + G + F, namely, the LG [55] amino acid substitution matrix, Gamma distribution (under four rate categories), and empirical amino acid frequencies, as the suitable model for the given alignment of UPOs and CPOs. A bootstrapped maximum likelihood (ML) tree was constructed with 1000 replicates using the recommended model. The LBA analysis was done using Tree puzzle 5.3 [56].

### Selection analyses

The selection analysis was carried out on the Datamonkey web server [57] of HyPhy package [58]. Branch-specific and site-specific methods were applied to UPO and CPO sequences. Fast, Unconstrained Bayesian AppRoximation (FUBAR) [28], Mixed Effects Maximum Likelihood (MEME) [27],was used to identify pervasive and episodic selective pressure on each site respectively, and adaptive branch-site random effects likelihood (aBSREL) [30] was used as the branch-site model, and Branch-site unrestricted statistical test for episodic diversification (BUSTED) [26] was used to identify gene-wide test for positive selection on the entire phylogeny of UPOs and CPOs separately instead of selecting a few branches. This method tests for positive selection whether a gene has experienced at at least one site on at least one branch (ω_1_ ≤ ω_2_ ≤ 1 ≤ ω_3_) [26]. It applies the unconstrained model to estimate the proportion of sites per partition belonging to each ω class and then the constrained model by comparing the former model to a null model where ω_3_ = 1, which means it disallows positive selection on the foreground branches. These methods allow heterogeneous nonsynonymous (dN) to synonymous (dS) rate ratios (ω = dN/dS) among the branches and across sites.

### Functional divergence analysis

Type-I and Type-II functional divergence of the Basidiomycota and Ascomycota UPOs, along with CPOs was estimated in pairs using DIVERGE 3.0 [59] at a bootstrapping value of 100. Type-I functional divergence coefficient (Ө_I_) identifies the sites having different evolutionary rates caused by the functional divergence [60] and Type-II functional divergence coefficient (Ө_II_) identifies the radical amino acid changes at some sites caused by the rapid evolution [61]. Another Type-I Analysis [62] which provides site-specific profile and residues involved in functional divergence, was performed for the five non-degenerate patterns, S0 to S4, where S0 means no Type-I divergence in any cluster, S1 signifies Type-I divergence in cluster 1, and so on, finally S4 means all the clusters have experienced Type-I divergence.

## Additional files

Additional file 1: **Table S1** Number of putative sequences obtained in 35 different fungal species using the pipeline. (DOCX 13 kb)

Additional file 2: **Figure S1** MSA of all UPO-encoding sequences and CPO sequences used as the outgroup. *Red* arrows point to PCP motif, *green* arrows point to EGD motif in UPOs, and *blue* arrows point to the acid-base catalyst pair in all UPOs. The asterisks (*) represent the conserved sites and # represents the clipped gapped sites in the alignment. (TIF 68781 kb)

Additional file 3: **Table S2** The binding cavity analysis of all the predicted structures of newly found UPOs. The binding pockets are shown in surface and the aromatic residues are shown in sticks. (DOCX 13720 kb)

Additional file 4: **Figure S2** MSA of the five different subfamilies of UPOs and newly found motifs highlighted with rectangles: *sky blue* for Subfamily-I, *pink* for Subfamily-II, *green* for Subfamily-III, *blue* represents motifs in Subfamily-IV, *yellow* represents motifs in Subfamily-V and *red* for the motifs found in all UPOs. (TIF 27409 kb)

Additional file 5: Selection analyses data for UPOs. (XLSX 119 kb)

Additional file 6: Selection analyses data for CPOs. (XLSX 109 kb)

Additional file 7: **Figure S3** MSA of all UPOs showing the positive and negatively selected sites using the MEME and FUBAR method. The asterisks (*) represent the conserved sites and the arrows point towards the motifs: PCP-EGD-R---E. (TIF 25856 kb)

Additional file 8: **Figure S4** A graph showing the number of (a) synonymous and (b) nonsynonymous sites in UPOs obtained using the MEME method. (TIF 649 kb)

Additional file 9: **Figure S5** Selection analysis on UPOs using aBSREL, a branch-site model. Thicker branches have a *p*-value < 0.05 showing evidence of undergoing positive diversifying selection. (TIF 1225 kb)

Additional file 10: **Figure S6** MSA of all CPOs showing the positive and negatively selected sites using the MEME and FUBAR method. The asterisks (*) represent the conserved sites and the arrows point towards the motifs: PCP-EHD---E. (TIF 17764 kb)

Additional file 11: **Figure S7** A graph showing the number of (a) synonymous and (b) nonsynonymous sites in CPOs obtained using the MEME method. (TIF 602 kb)

Additional file 12: **Figure S8** Selection analysis on CPOs using aBSREL, a branch-site model. Thicker branches have a p-value < 0.05 showing evidence of positive diversifying selection. (TIF 1002 kb)

Additional file 13: **Figure S9** An MSA of the clusters formed for the functional divergence analysis showing the Type-I functional divergent sites highlighted with *black* color. (TIF 7611 kb)

Additional file 14: Functional divergence analysis data for UPOs and CPOs. (XLSX 77 kb)

Additional file 15: **Figure S10** Structural representation of the newly found motifs adhering near the binding pockets (shown as surface) are shown in one species from each subfamily of UPOs; a) experimentally resolved structure of *Aae*UPO and modeled structures of b) *Mixia osmundae iam14324,* c) *Jaapia argillacea mucl33604,* d) *Kalmanozyma brasiliensis ghg001,* e) *Glarea lozoyensis atcc20868,* and f) *Phialocephala scopiformis. (TIF 2498 kb)*

Additional file 16: **Table S3** Information of all the fungal genome sequences used in this study. (XLSX 38 kb)

Additional file 17: **Table S4** CPO sequences and *Mro*UPO used in this study. (DOCX 14 kb)

## Abbreviations

*Aae*: *Agrocybe aegerita*
CPO: Chloroperoxidase
LBA: Long branch attraction
*Lfu*: *Leptoxyphium fumago*
*Mro*: M*arasmius rotula*
MSA: Multiple Sequence Alignment
Pog: Peroxidase-peroxygenase
UPO: Unspecific peroxygenase
