# Supplementary material for: New insights on unspecific peroxygenases: superfamily reclassification and evolution
Source: BMC Evol Biol. 2019 Mar 13;19:76. doi: 10.1186/s12862-019-1394-3 (PMC6417270; doi:10.1186/s12862-019-1394-3)
Supplement: Supplementary file 1 — Table S1 Number of putative sequences obtained in 35 different fungal species using the pipeline. (DOCX 13 kb) [file 12862_2019_1394_MOESM1_ESM.docx]

| Fungal species | Number of putative sequences |
| --- | --- |
| *Exidia glandulosa hhb12029* | 9 |
| *Agaricus bisporus var burnettii jb137s8* | 10 |
| *Pseudozyma hubeiensis sy62* | 1 |
| *Hebeloma cylindrosporum h7* | 2 |
| *Sistotremastrum niveocremeum hhb9708* | 6 |
| *Jaapia argillacea mucl33604* | 3 |
| *Piloderma croceum f1598* | 2 |
| *Fibulorhizoctonia sp cbs109695* | 4 |
| *Trichosporon asahii var asahii cbs2479* | 3 |
| *Ustilago maydis* | 2 |
| *Ustilago hordei* | 1 |
| *Sphaerobolus stellatus ss14* | 26 |
| *Coprinopsis cinerea okayama7.130* | 5 |
| *Trichosporon asahii var asahii cbs8904* | 2 |
| *Galerina marginata cbs339.88* | 11 |
| *Cutaneotrichosporon oleaginosus* | 1 |
| *Hypholoma sublaterium fd334ss4* | 6 |
| *Sporisorium reilianum* | 1 |
| *Kalmanozyma brasiliensis ghg001* | 1 |
| *Glarea lozoyensis atcc20868* | 1 |
| *Aureobasidium melanogenum cbs110374* | 1 |
| *Mixia osmundae iam14324* | 1 |
| *Laccaria amethystine laam08.1* | 1 |
| *Zymoseptoria tritici* | 1 |
| *Paraphaeosphaeria sporulosa* | 1 |
| *Neonectria ditissima* | 1 |
| *Phialocephala scopiformis* | 1 |
| *Tilletia walkeri* | 2 |
| *Aureobasidium namibiae cbs147.97* | 1 |
| *Moesziomyces antarcticus* | 1 |
| *Acidomyces richmondensis* | 1 |
| *Tilletia controversa* | 1 |
| *Phialocephala subalpina* | 1 |
| *Sphaerulina_musiva_so2202* | 1 |
| *Sistotremastrum_suecicum_hhb10207ss3* | 1 |

**Additional Table S1** Number of putative sequences obtained in 35 different fungal species using the pipeline.
