# Supplementary material for: New insights on unspecific peroxygenases: superfamily reclassification and evolution
Source: BMC Evol Biol. 2019 Mar 13;19:76. doi: 10.1186/s12862-019-1394-3 (PMC6417270; doi:10.1186/s12862-019-1394-3)
Supplement: Supplementary file 3 — Table S2 The binding cavity analysis of all the predicted structures of newly found UPOs. The binding pockets are shown in surface and the aromatic residues are shown in sticks. (DOCX 13720 kb) [file 12862_2019_1394_MOESM3_ESM.docx]

**Additional Table S2** The binding cavity analysis of all the predicted structures of newly found UPOs. The binding pockets are shown in surface and the aromatic residues are shown in sticks.

| UPO | Predicted structure showing binding cavity |
| --- | --- |
| *Exidia glandulosa hhb12029* | 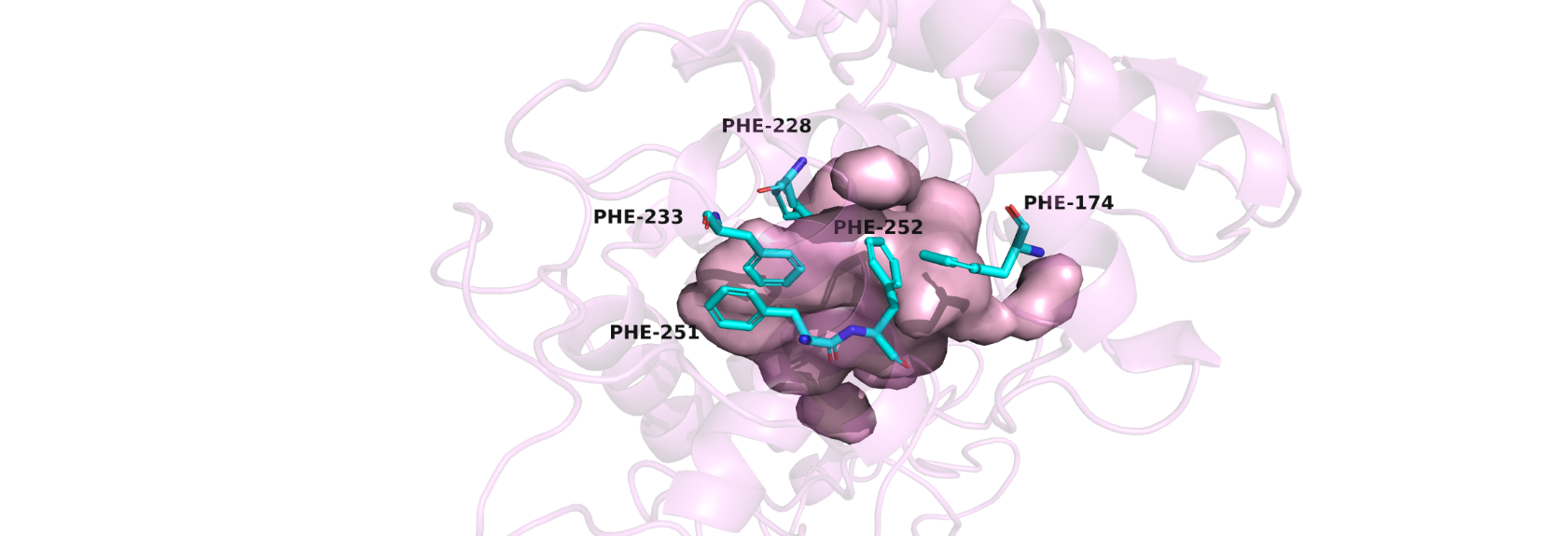 |
| *Agaricus bisporus var burnettii jb137s8* | 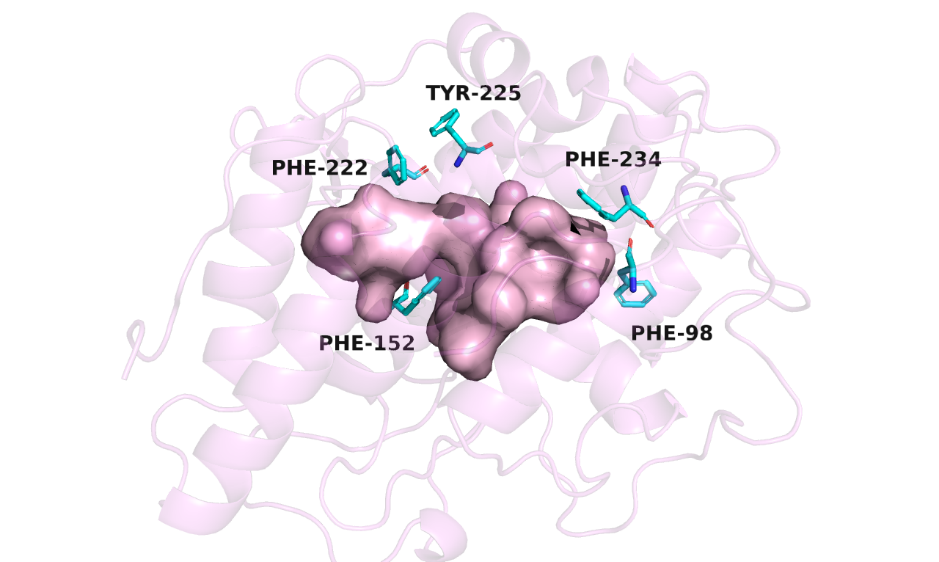 |
| *Pseudozyma hubeiensis sy62* | 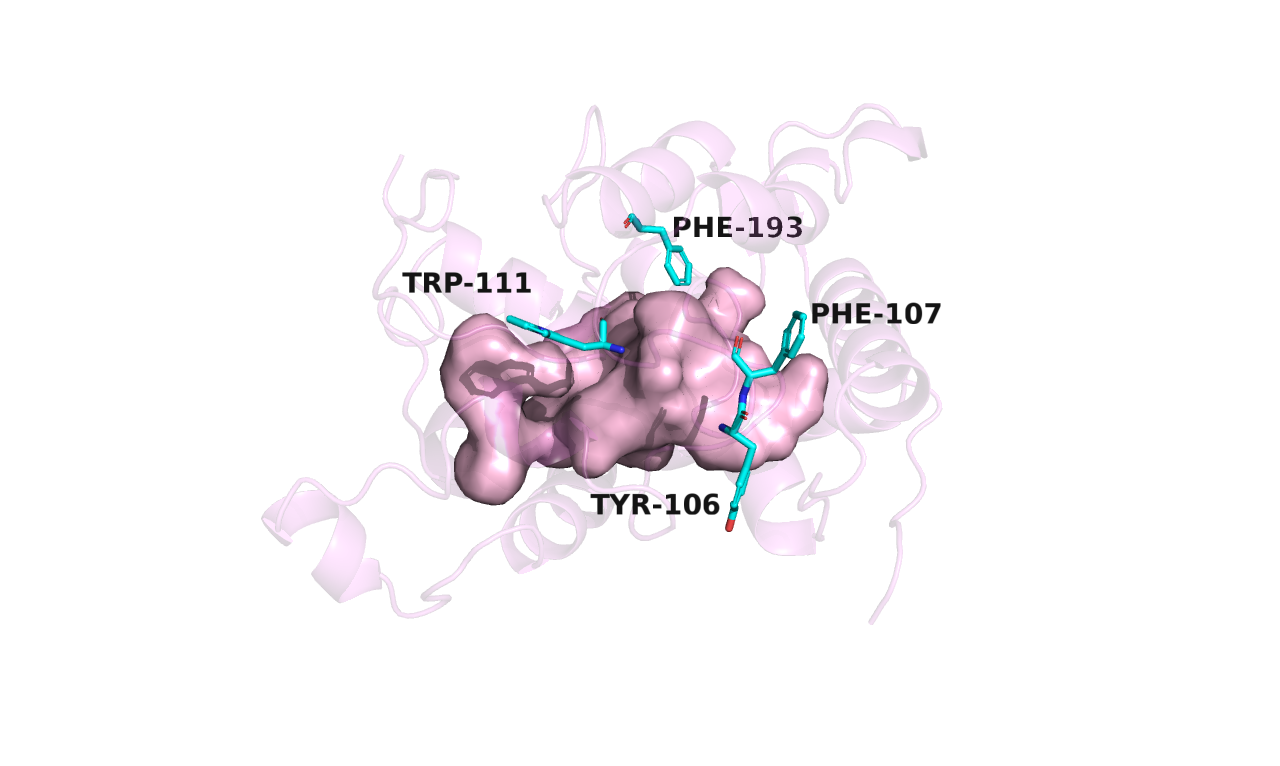 |
| *Hebeloma cylindrosporum h7* | 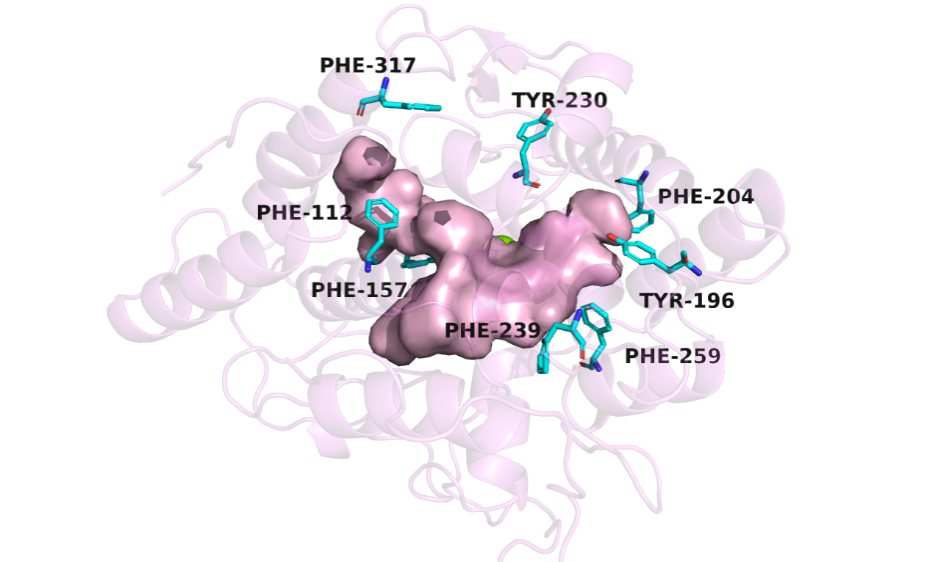 |
| *Sistotremastrum niveocremeum hhb9708* | 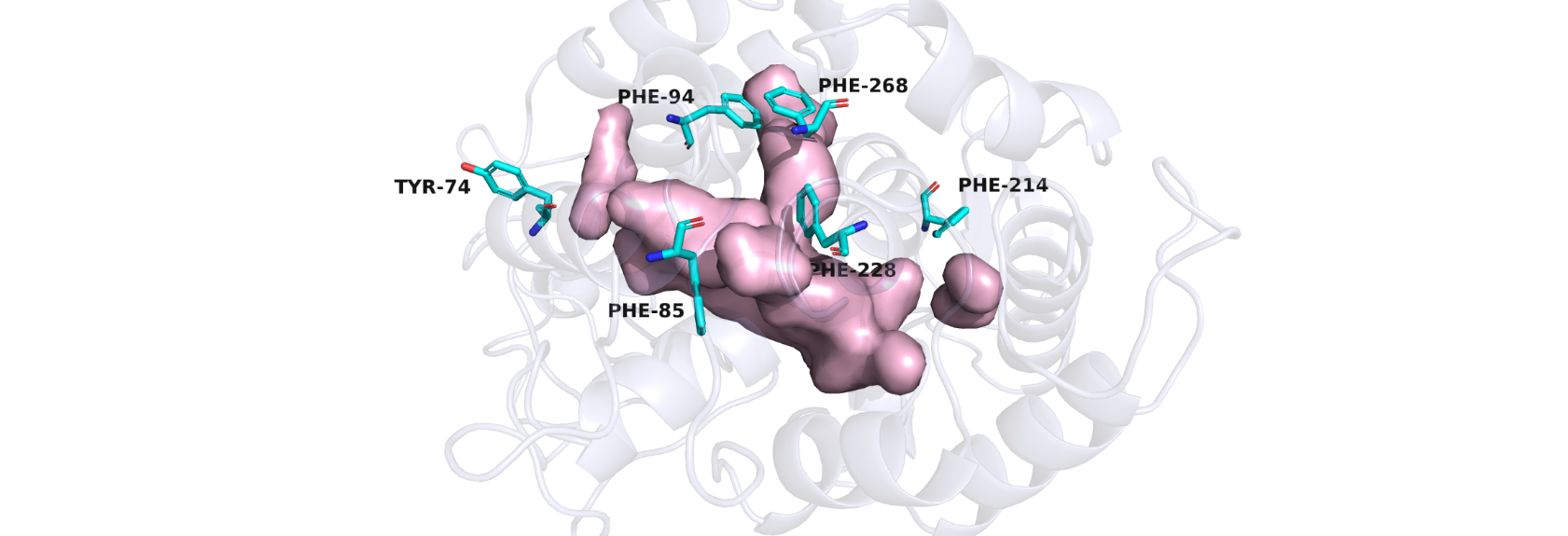 |
| *Jaapia argillacea mucl33604* | 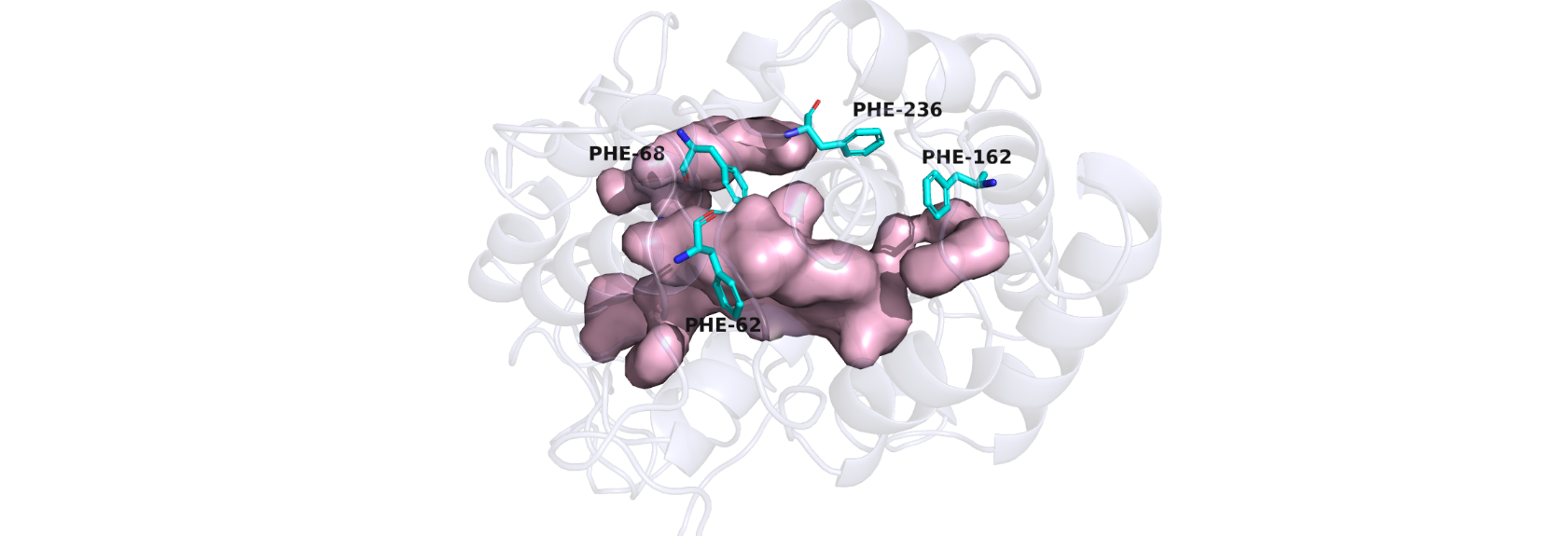 |
| *Piloderma croceum f1598* | 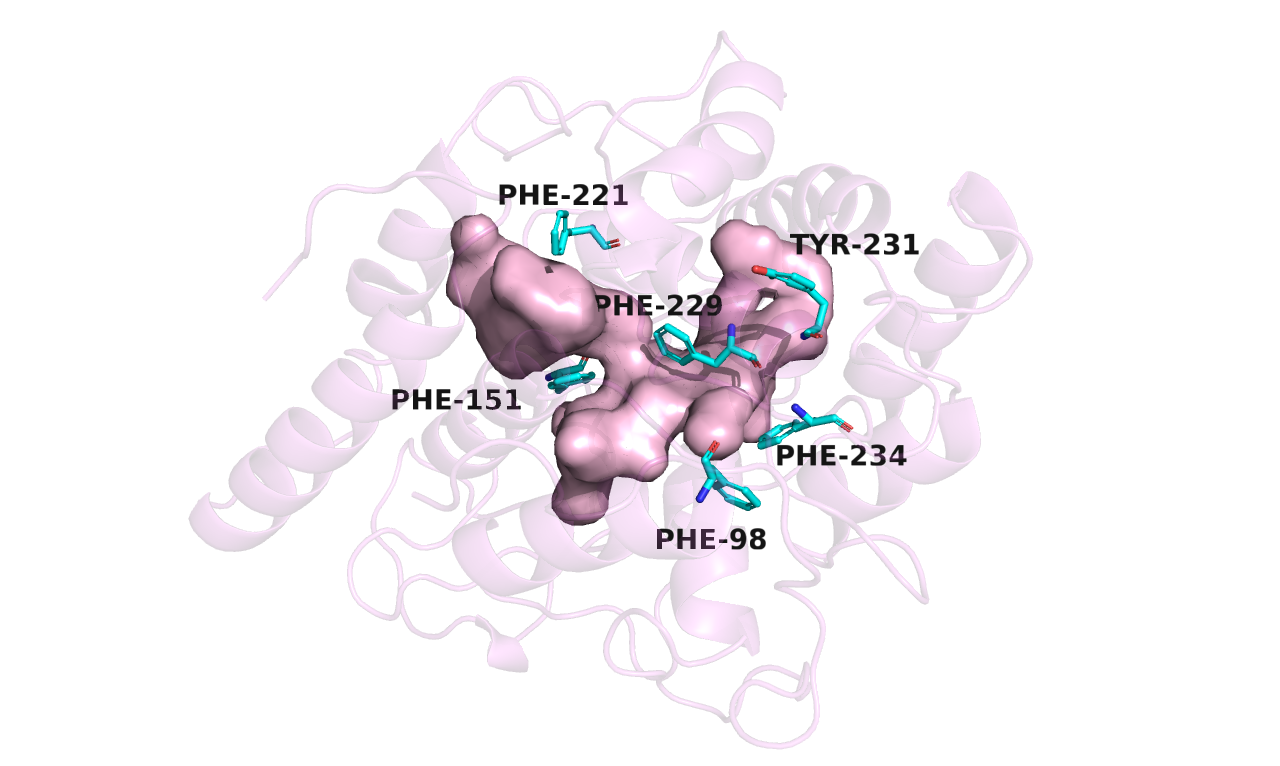 |
| *Fibulorhizoctonia sp cbs109695* | 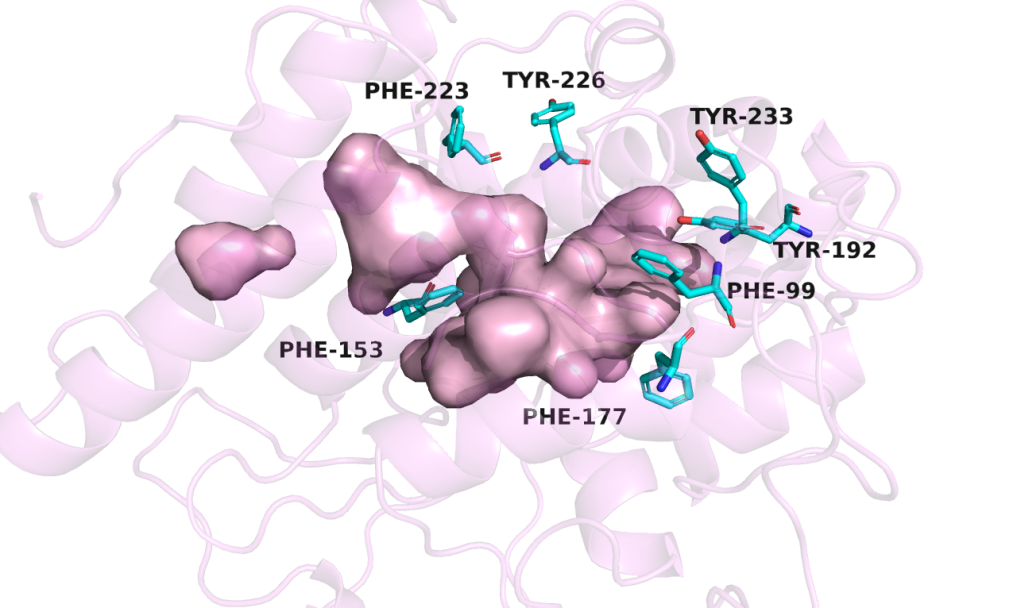 |
| *Trichosporon asahii var asahii cbs2479* | 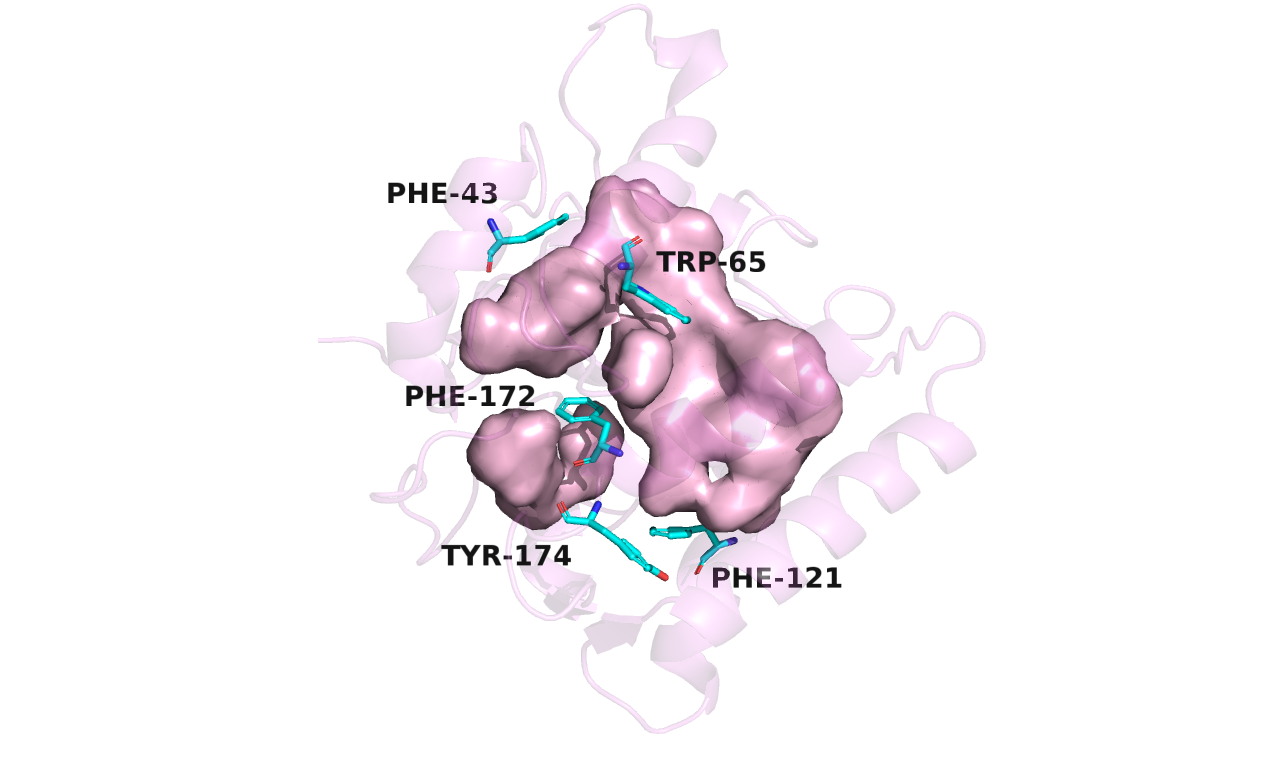 |
| *Ustilago maydis* | 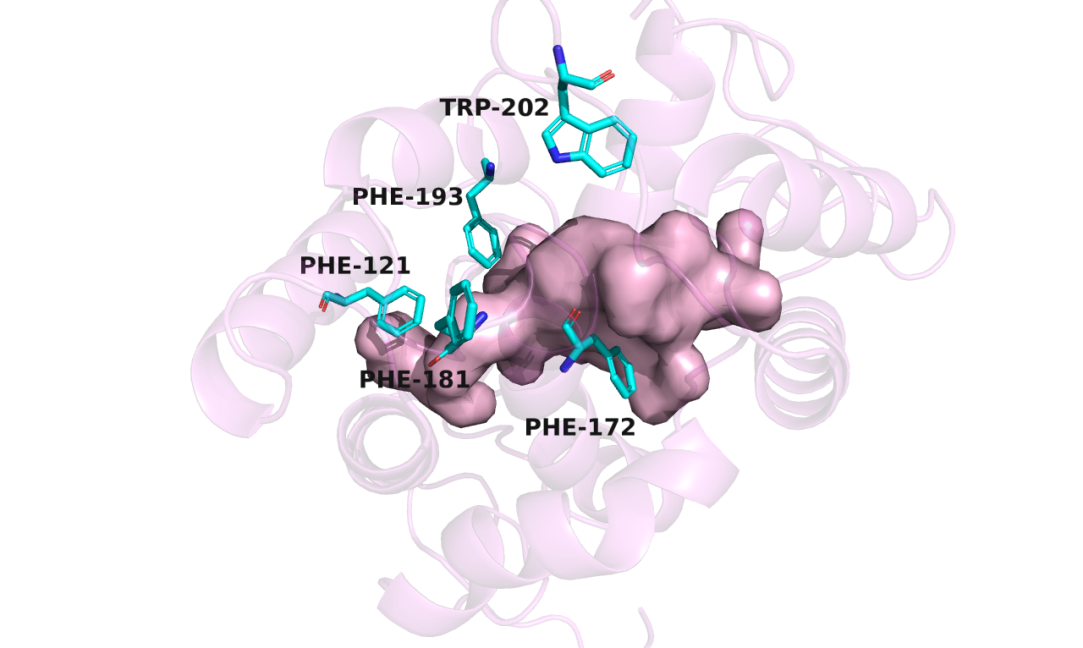 |
| *Ustilago hordei* | 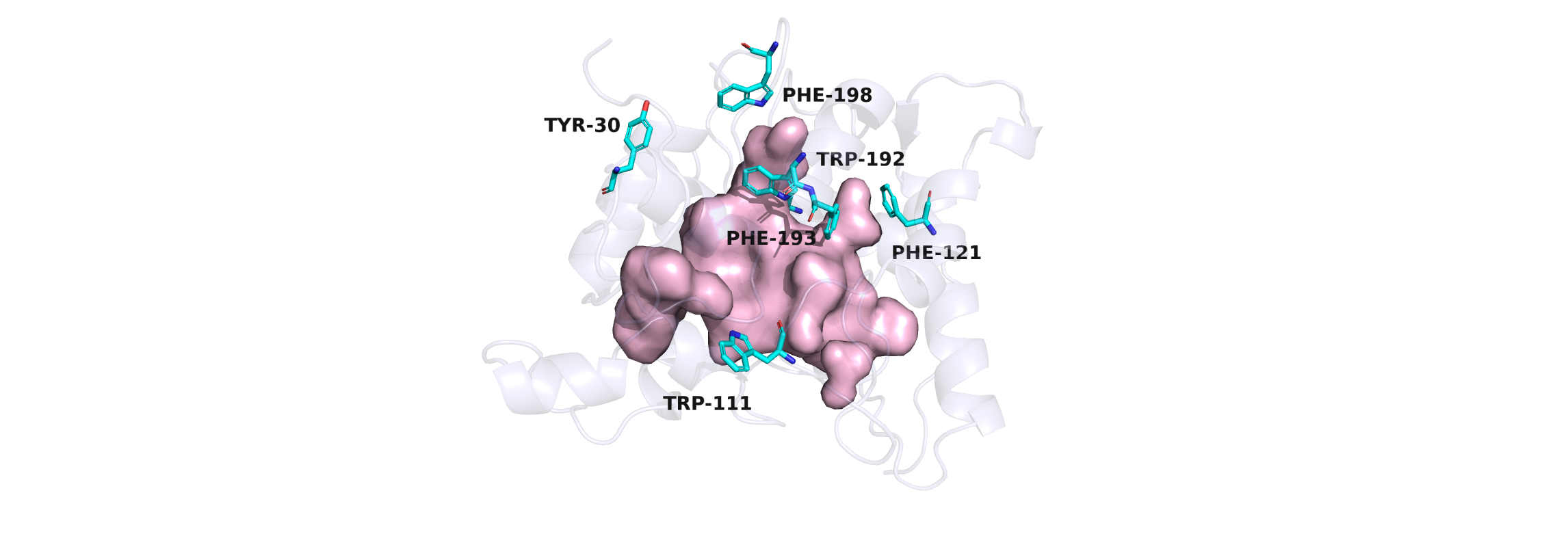 |
| *Sphaerobolus stellatus ss14* | 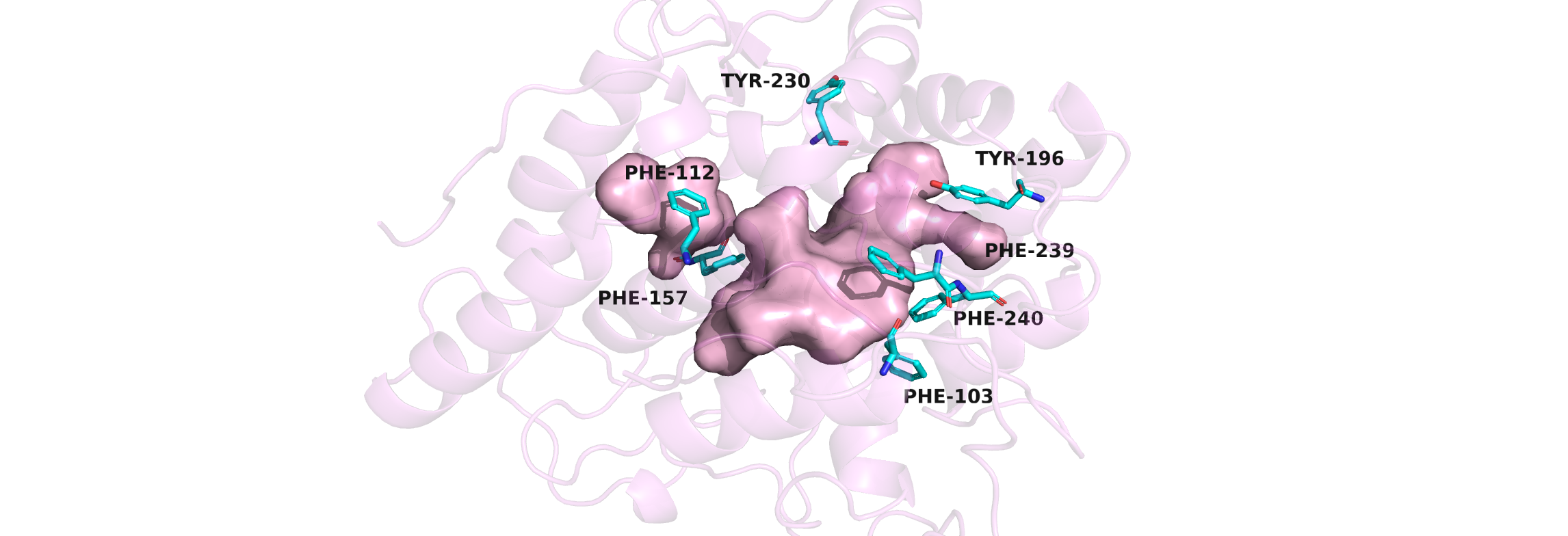 |
| *Coprinopsis cinerea okayama7.130* | 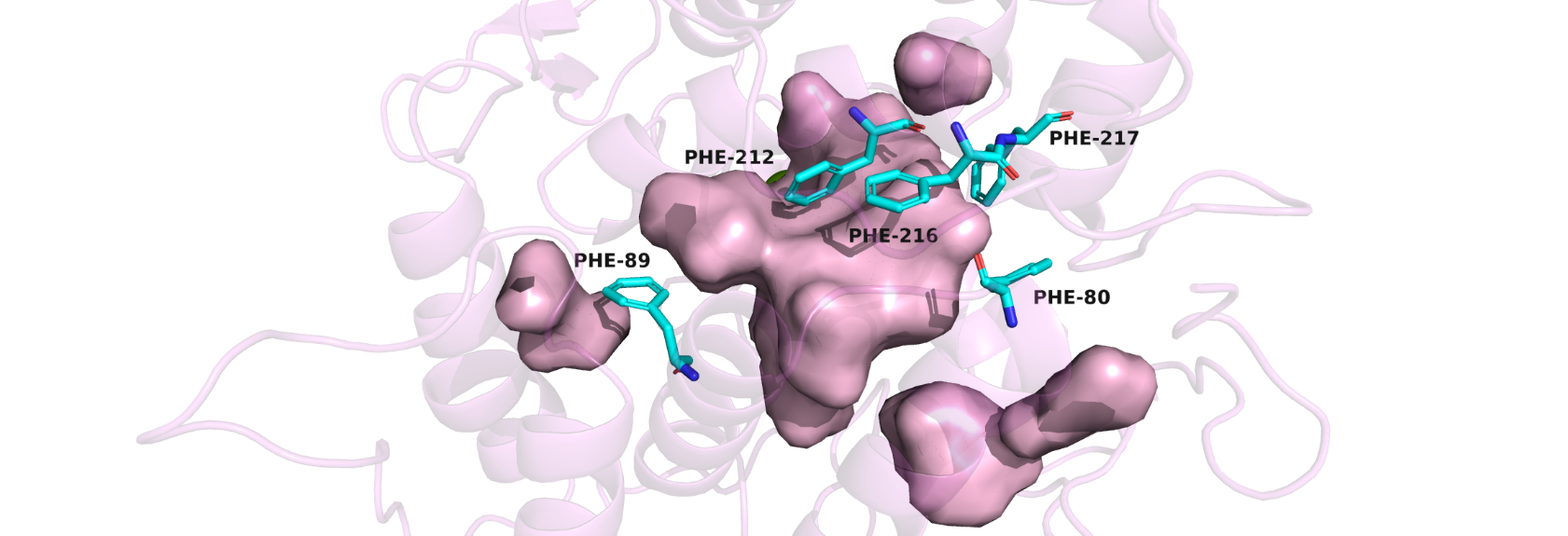 |
| *Trichosporon asahii var asahii cbs8904* | 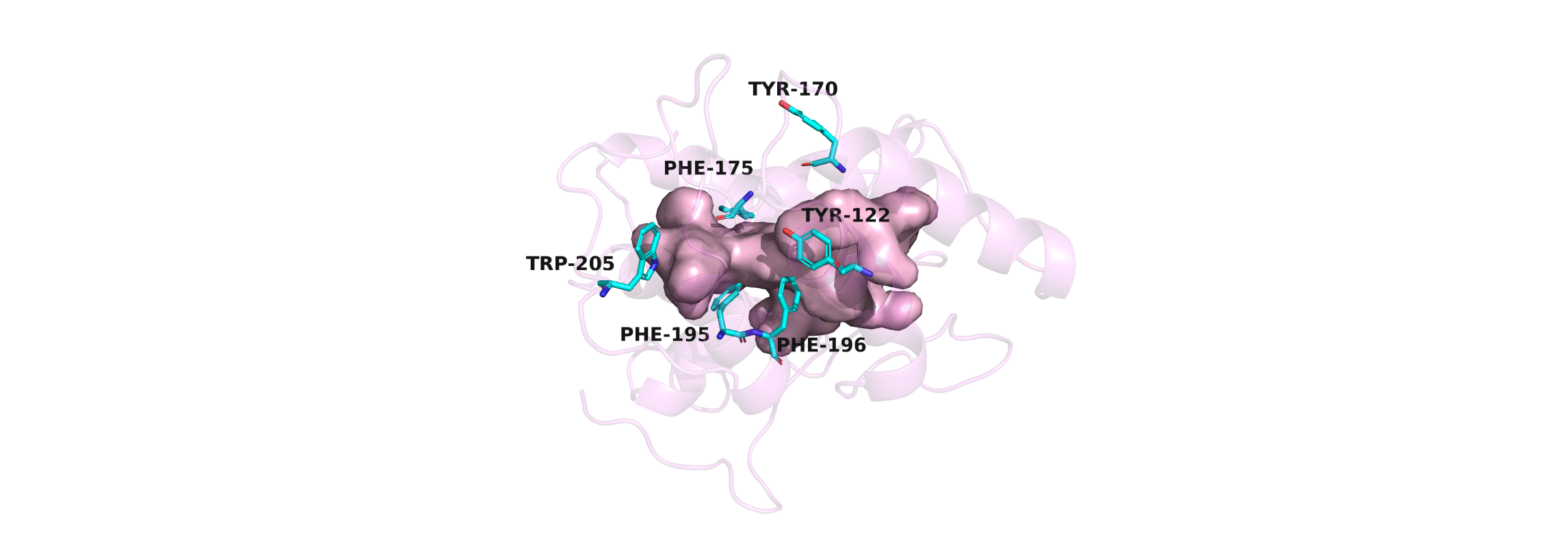 |
| *Galerina marginata cbs339.88* | 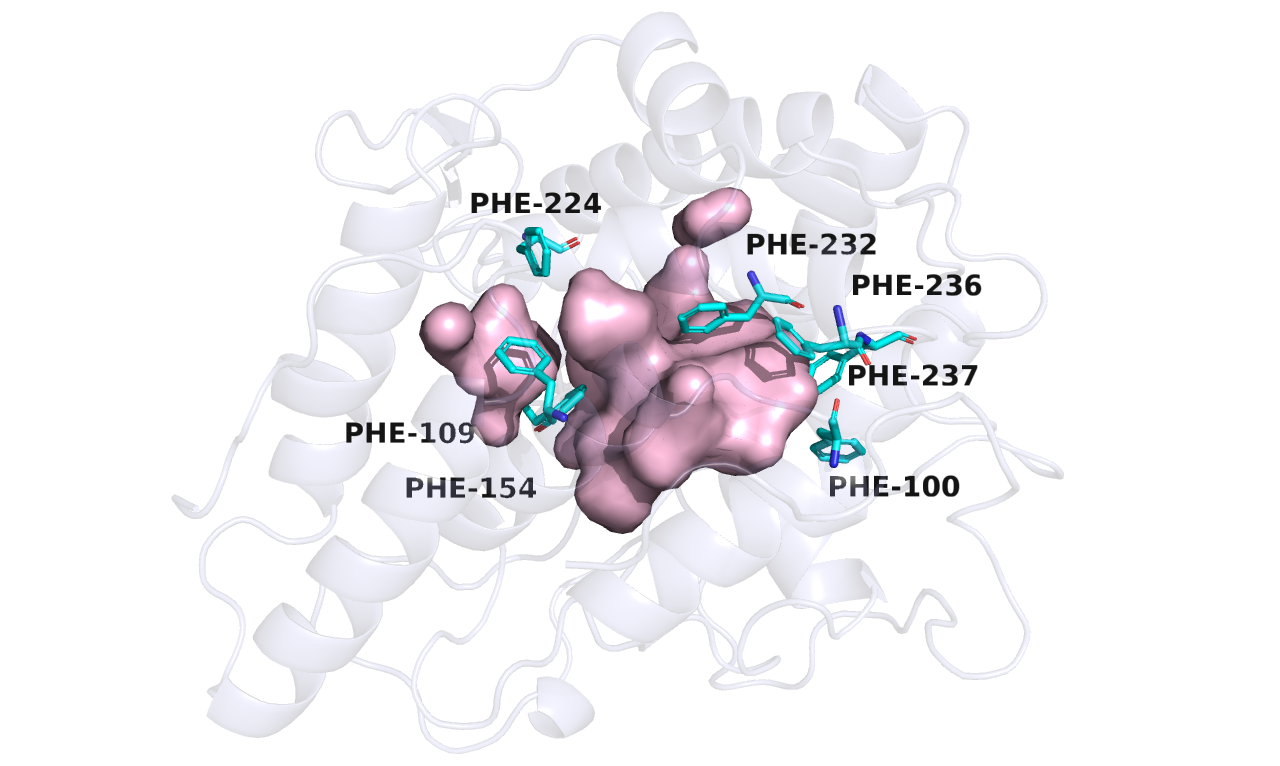 |
| *Cutaneotrichosporon oleaginosus* | 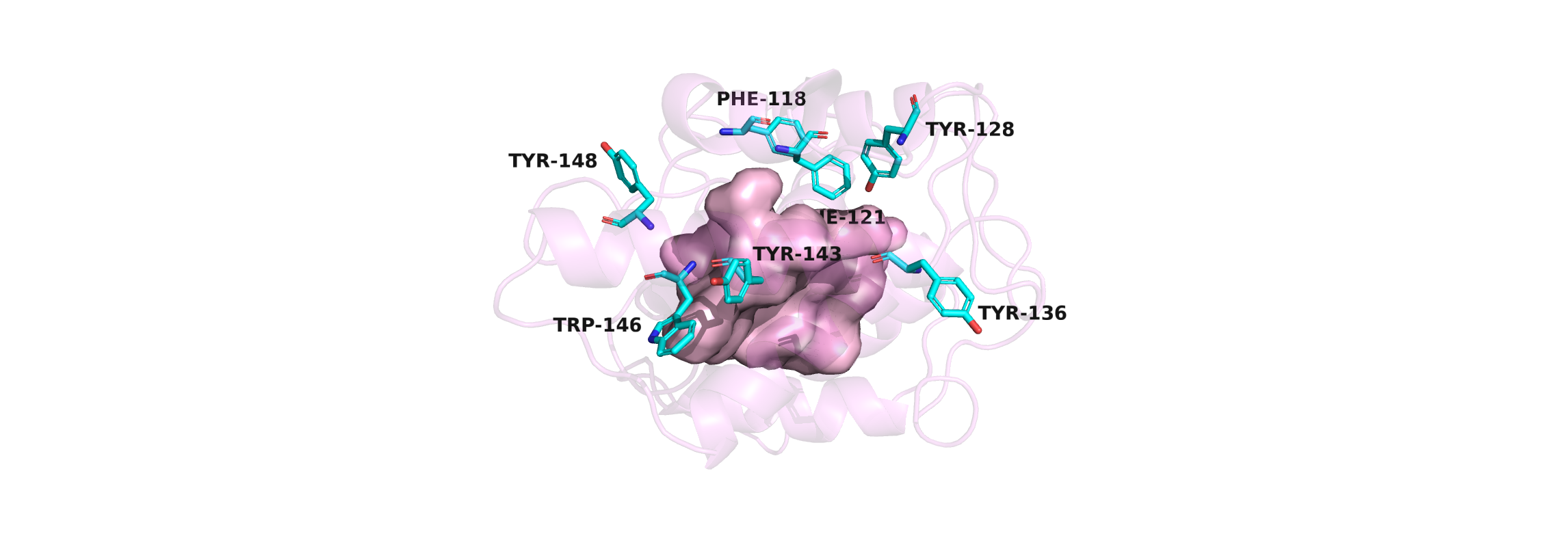 |
| *Hypholoma sublaterium fd334ss4* | 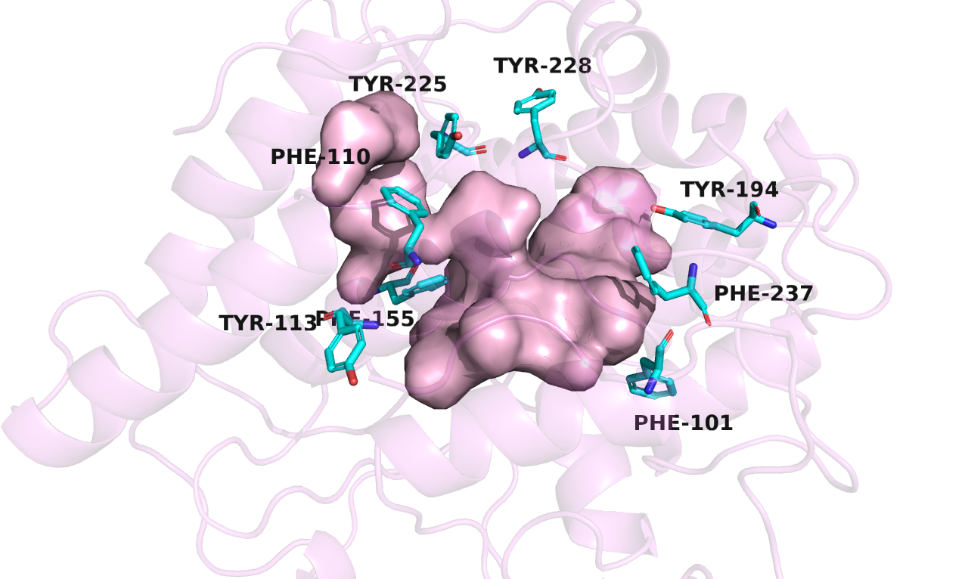 |
| *Sporisorium reilianum* | 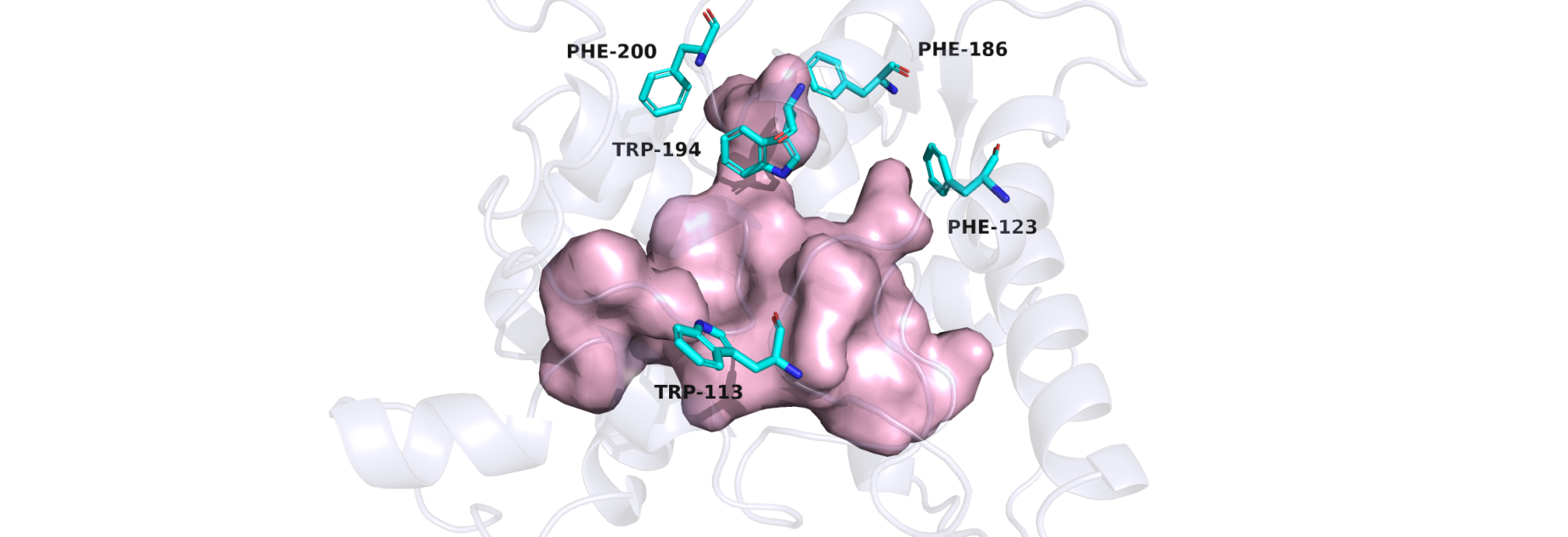 |
| *Kalmanozyma brasiliensis ghg001* | 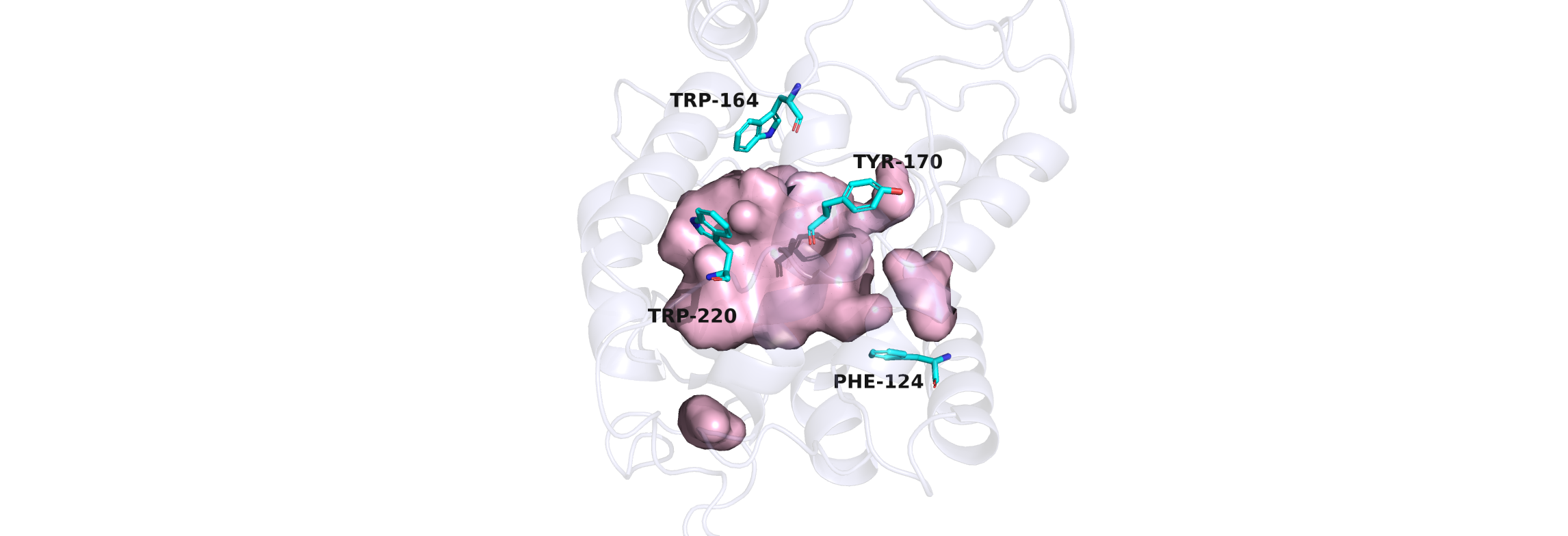 |
| *Glarea lozoyensis atcc20868* | 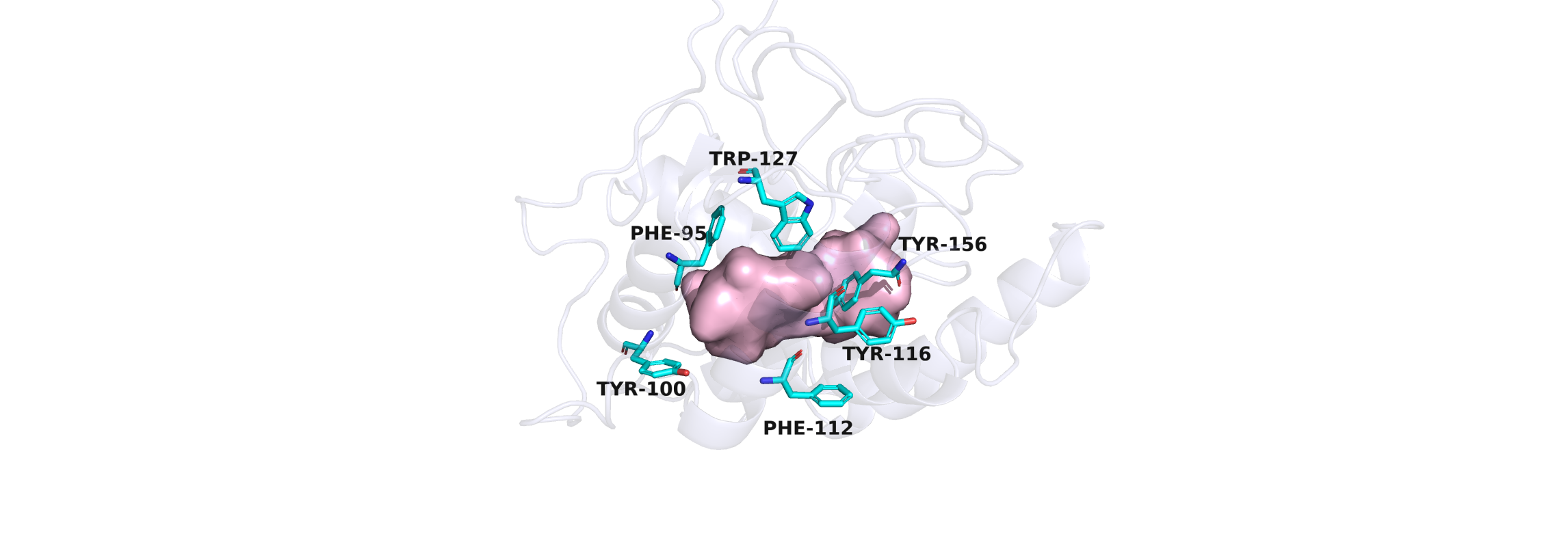 |
| *Aureobasidium melanogenum cbs110374* | 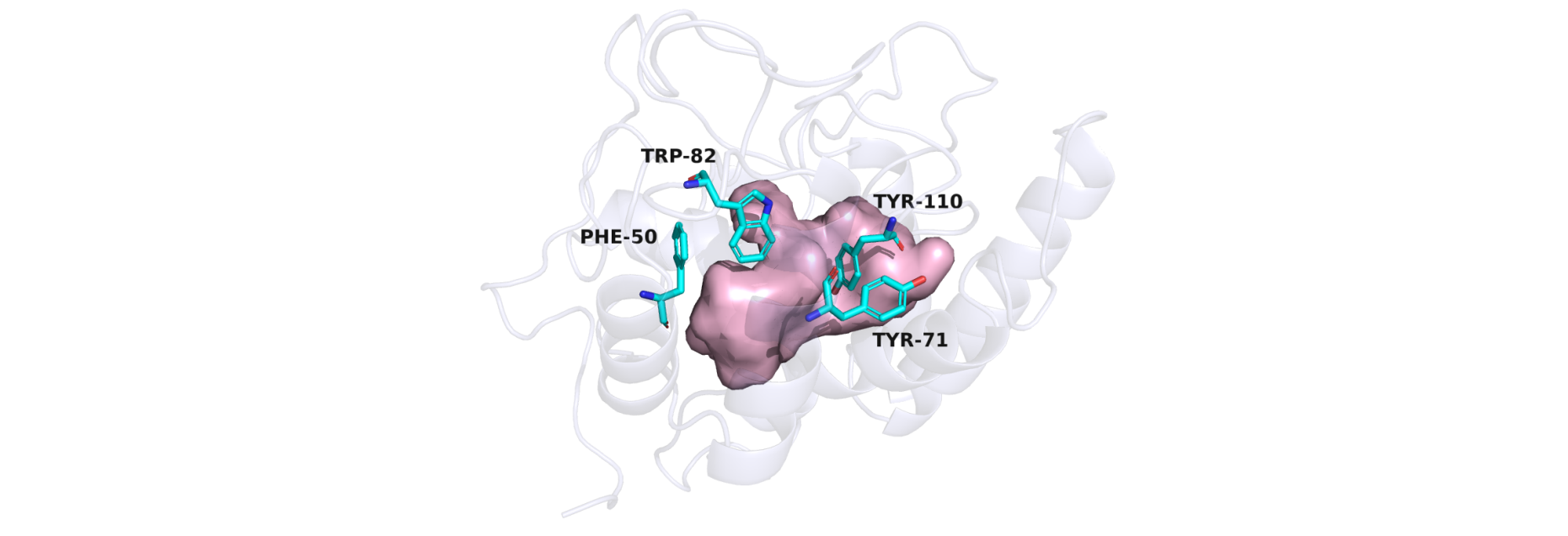 |
| *Mixia osmundae iam14324* | 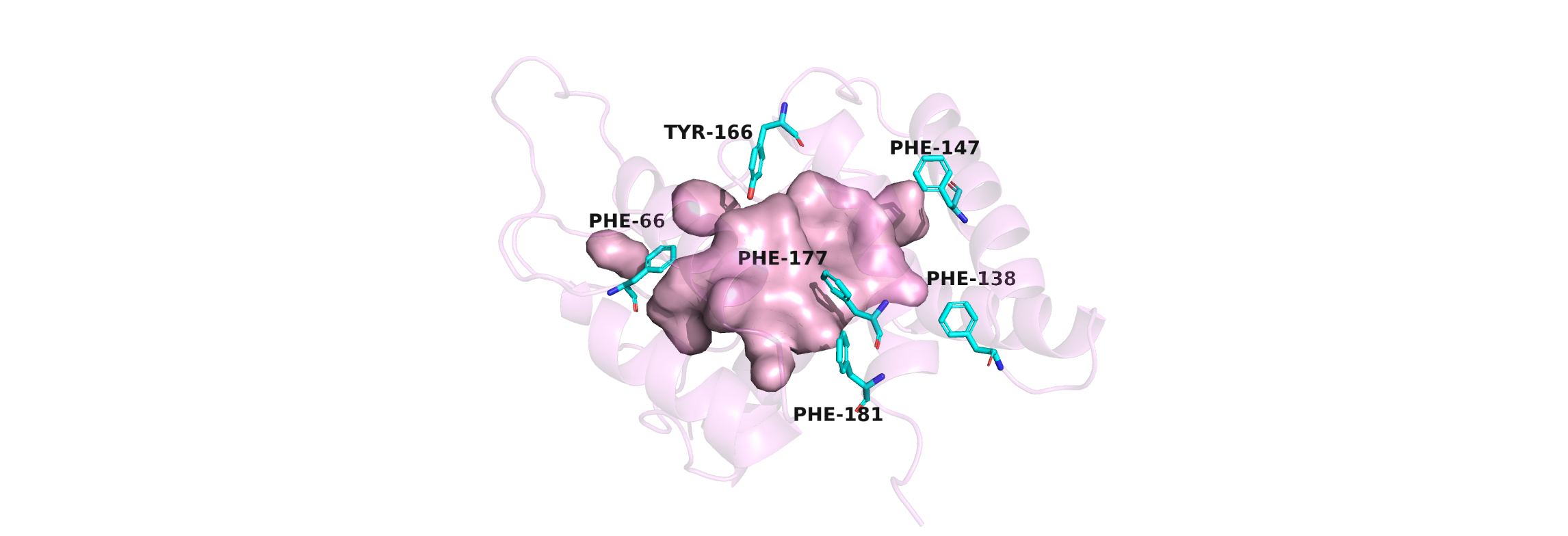 |
| *Laccaria amethystine laam08.1* | 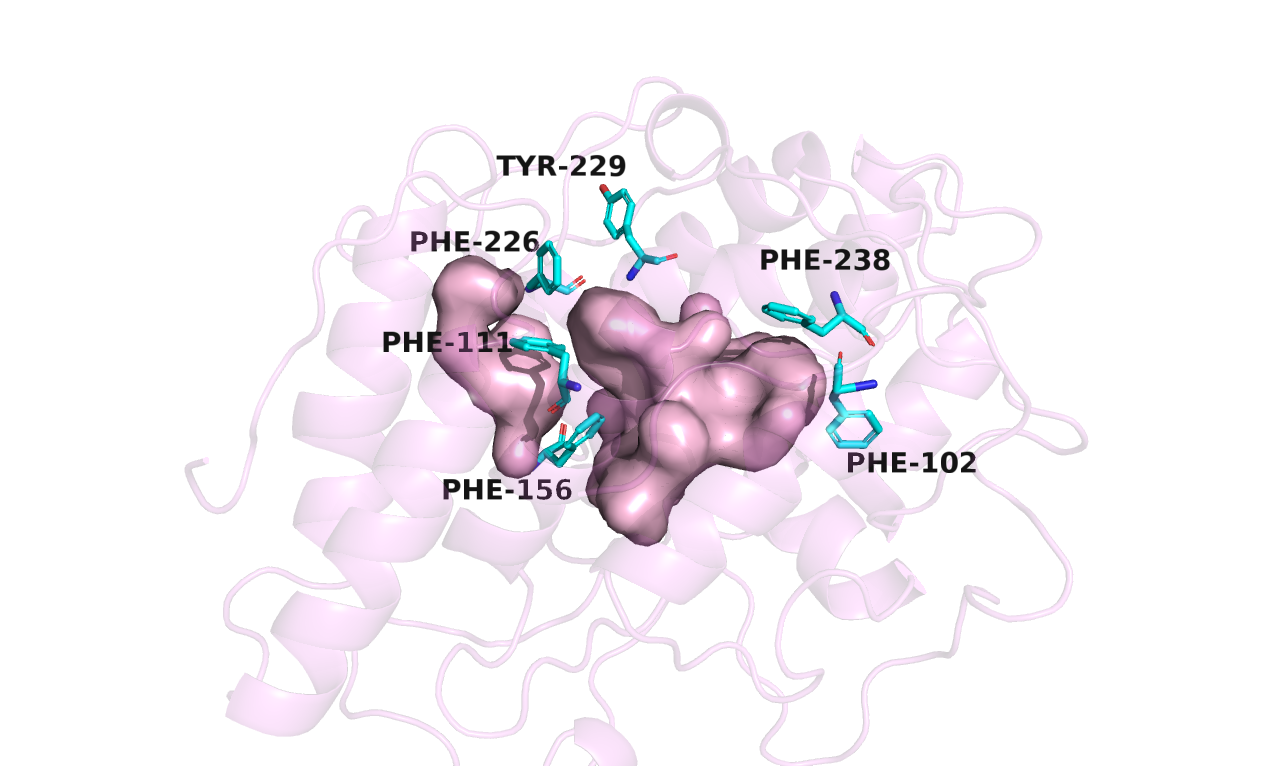 |
| *Zymoseptoria tritici* | 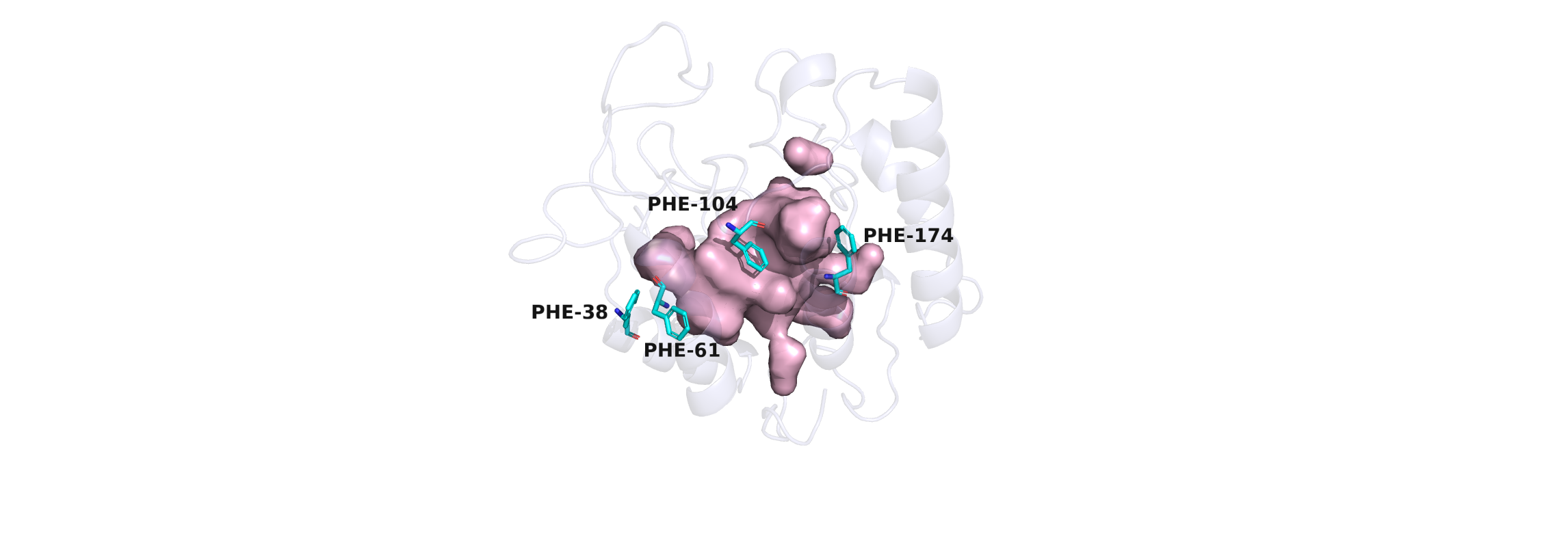 |
| *Paraphaeosphaeria sporulosa* | 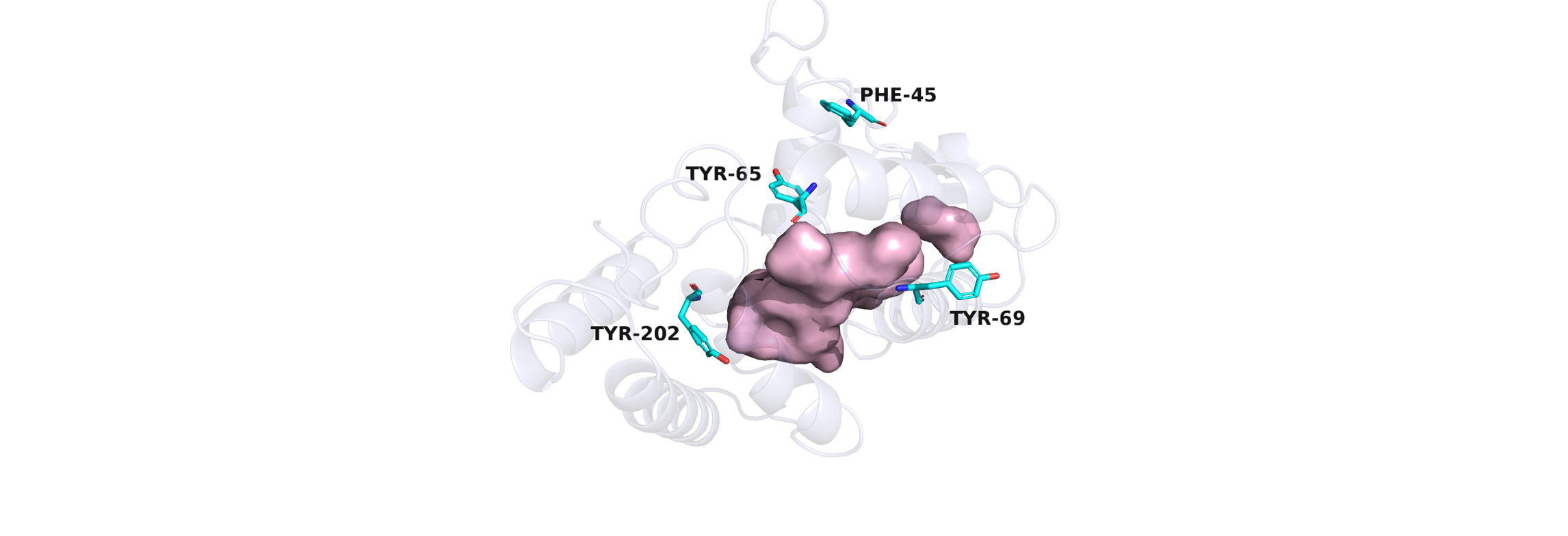 |
| *Neonectria ditissima* | 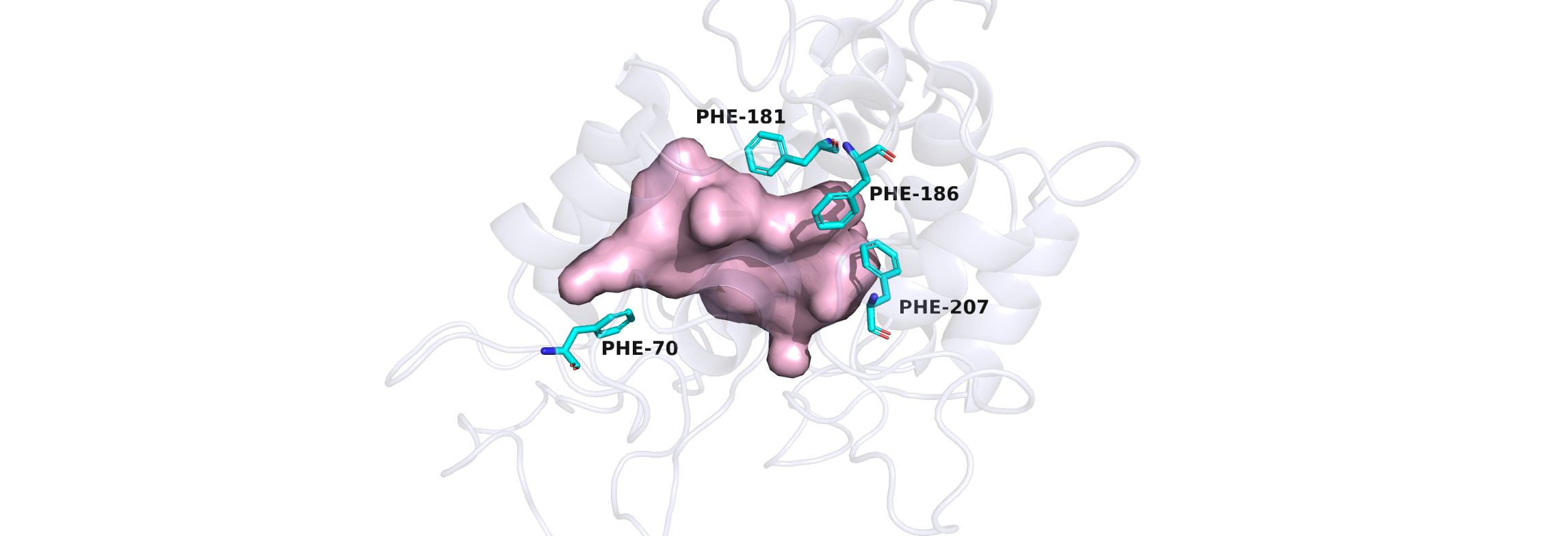 |
| *Phialocephala scopiformis* | 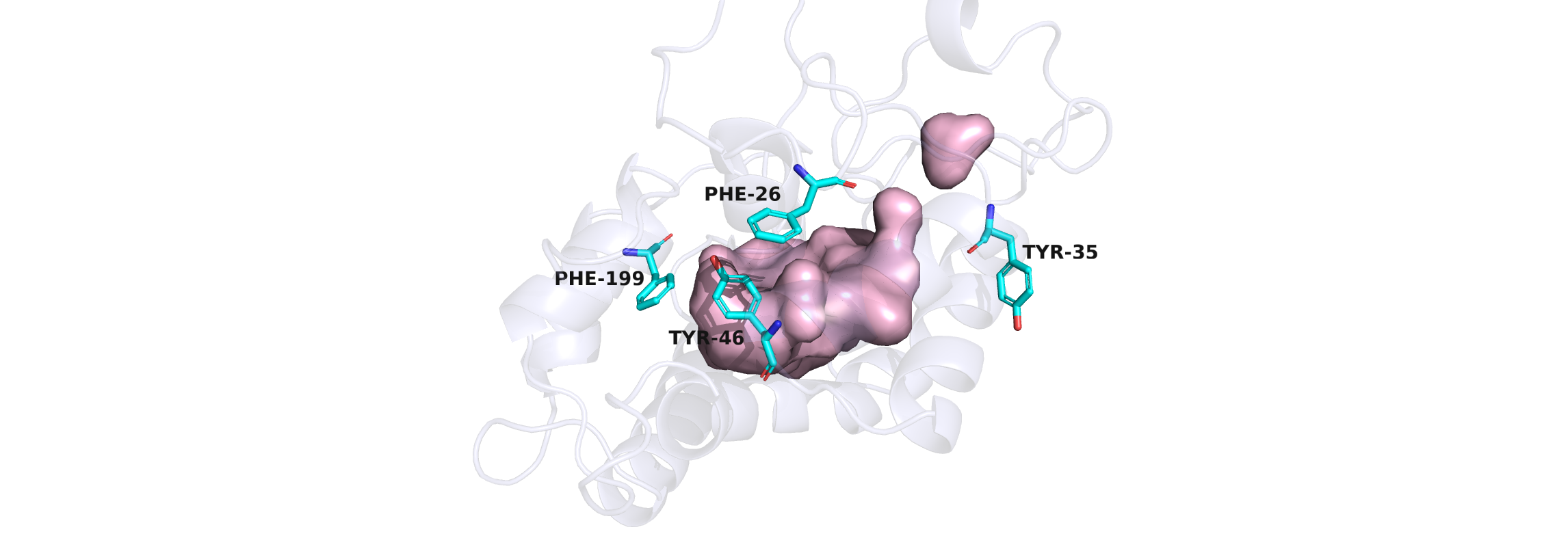 |
| *Tilletia walkeri* | 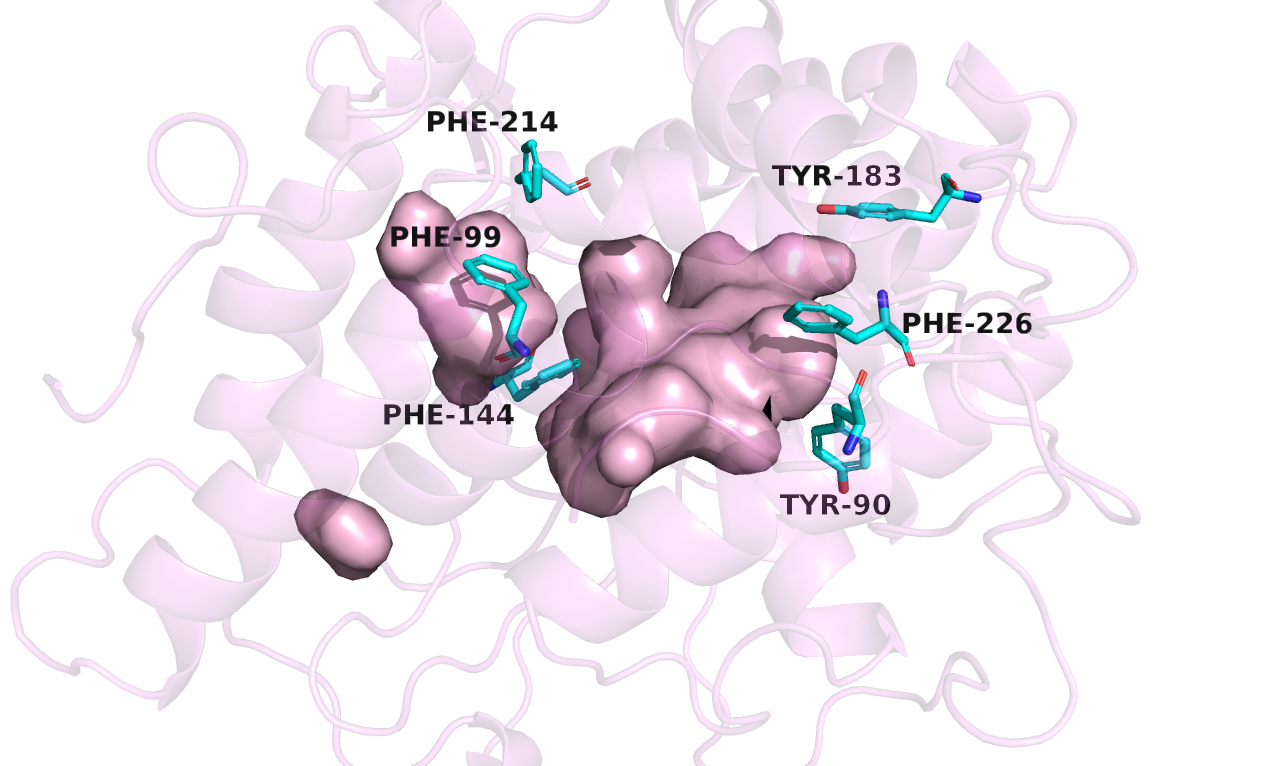 |
| *Aureobasidium namibiae cbs147.97* | 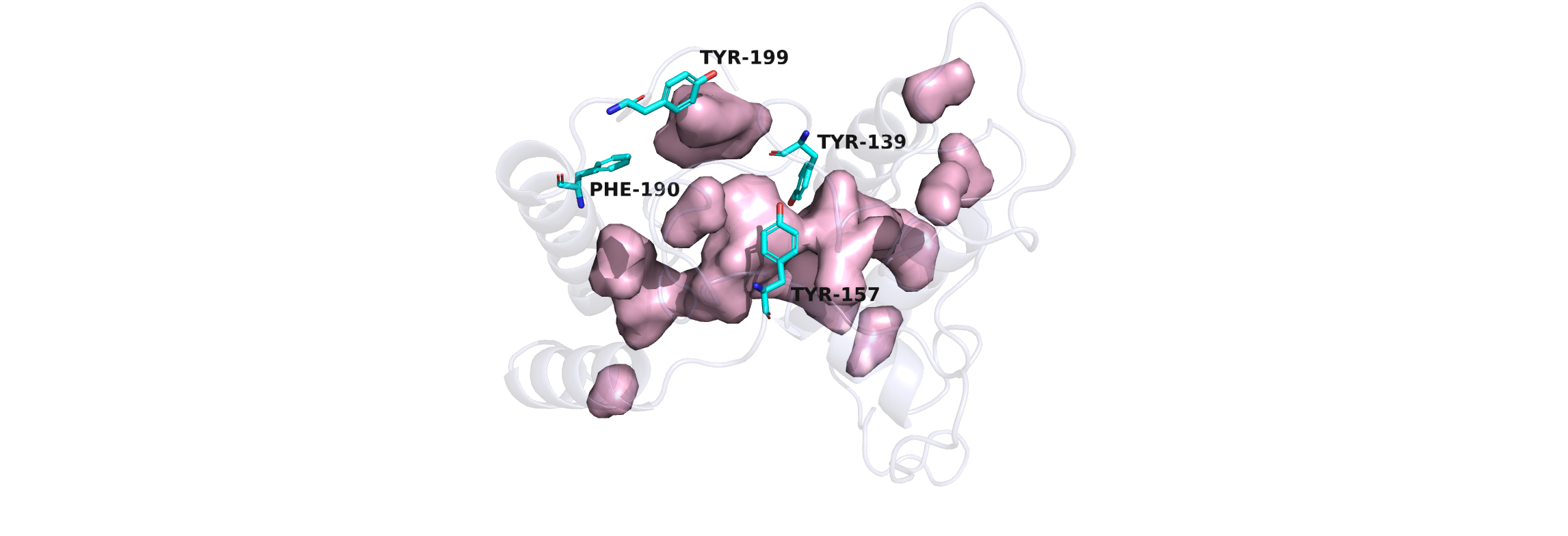 |
| *Moesziomyces antarcticus* | 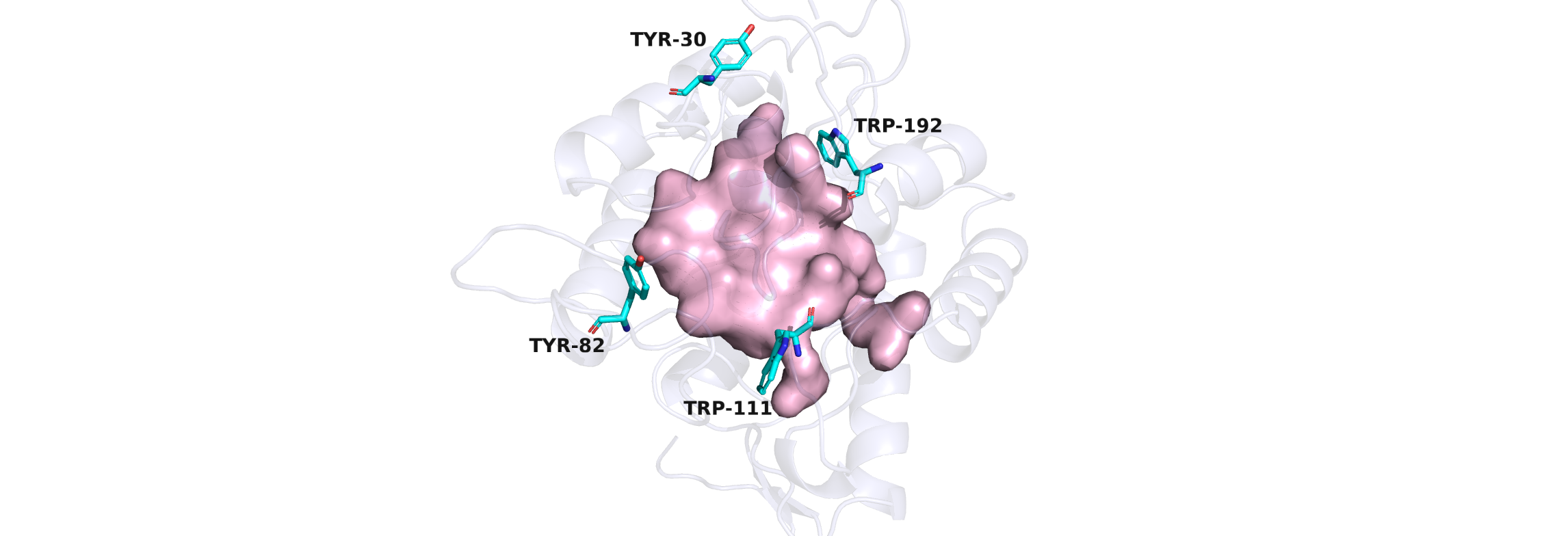 |
| *Acidomyces richmondensis* | 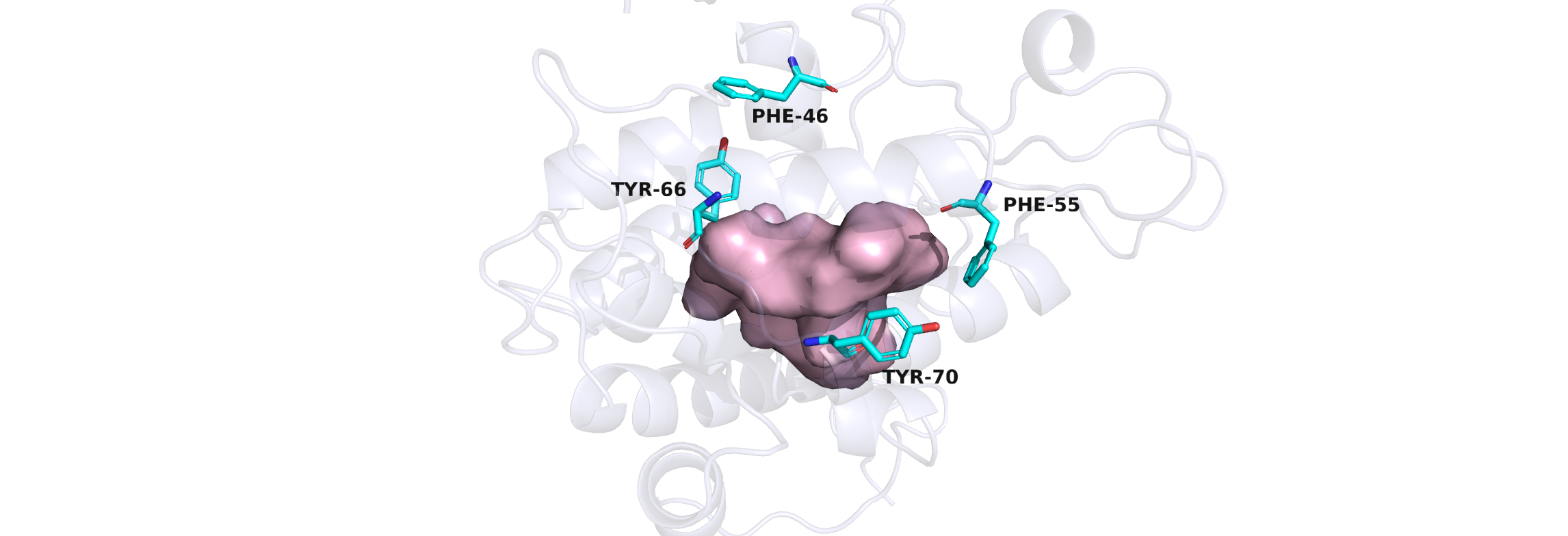 |
| *Tilletia controversa* | 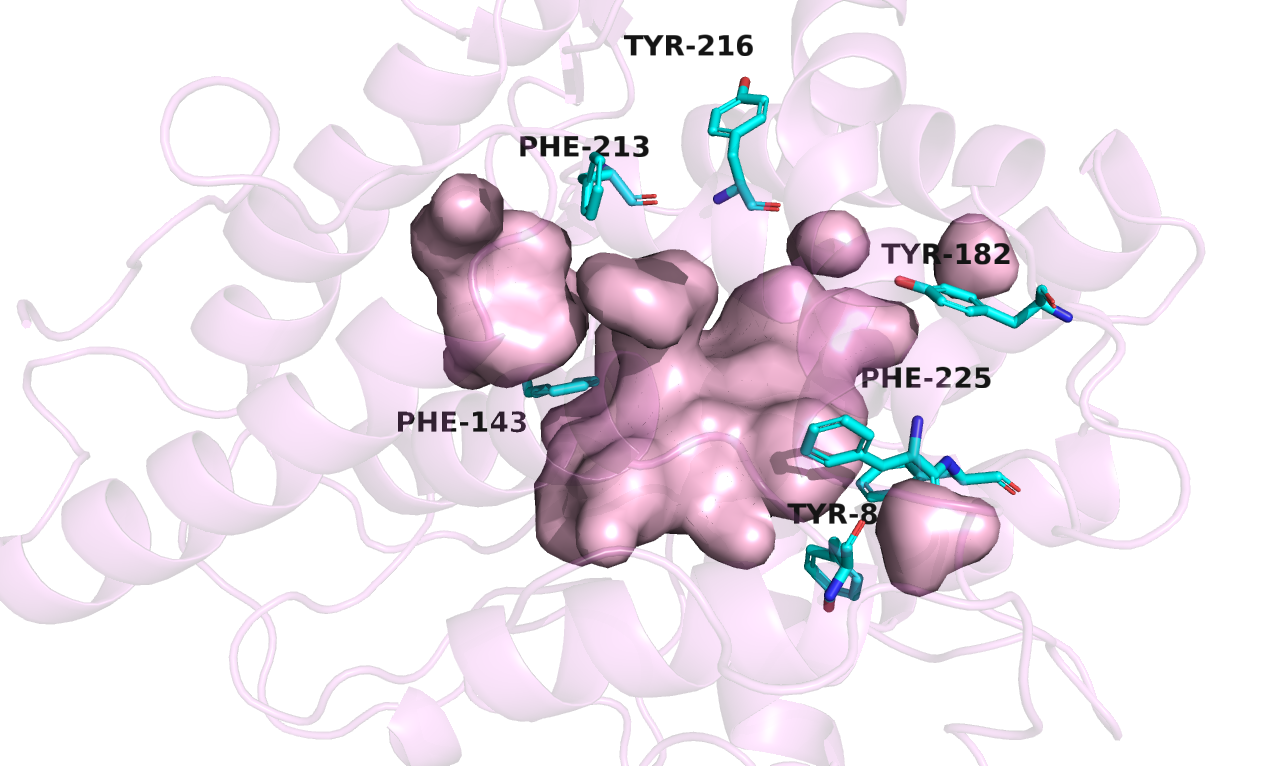 |
| *Phialocephala subalpina* | 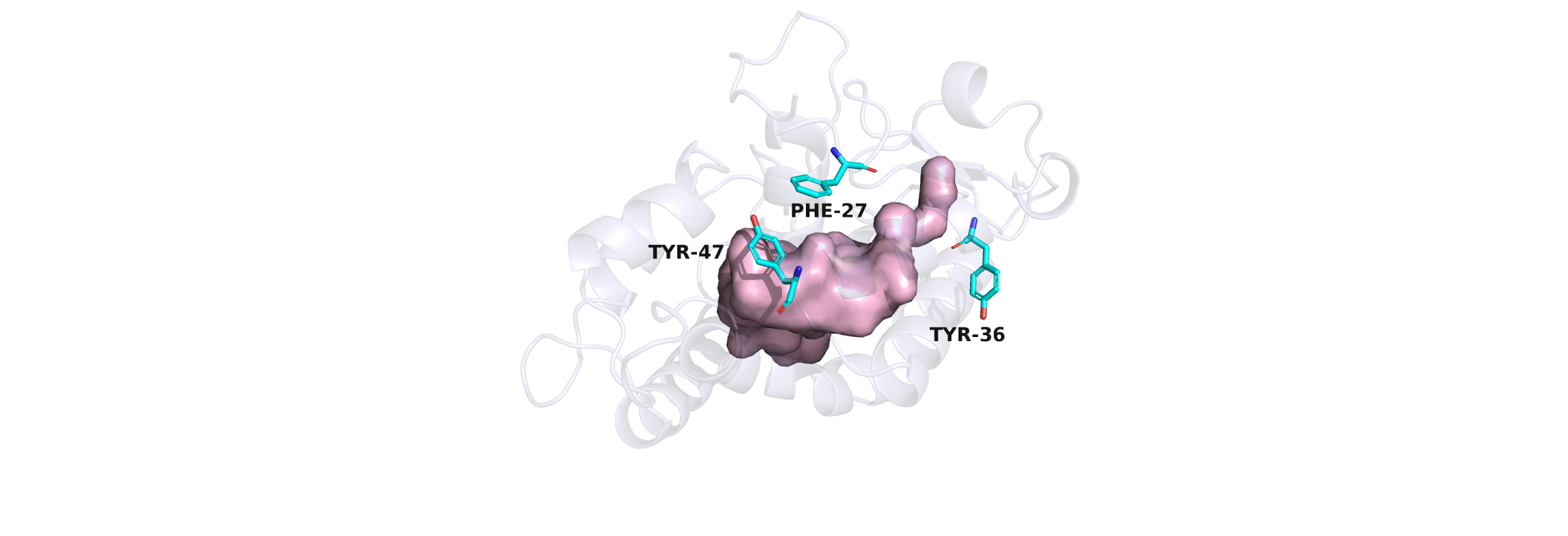 |
| *Sphaerulina musiva so2202* | 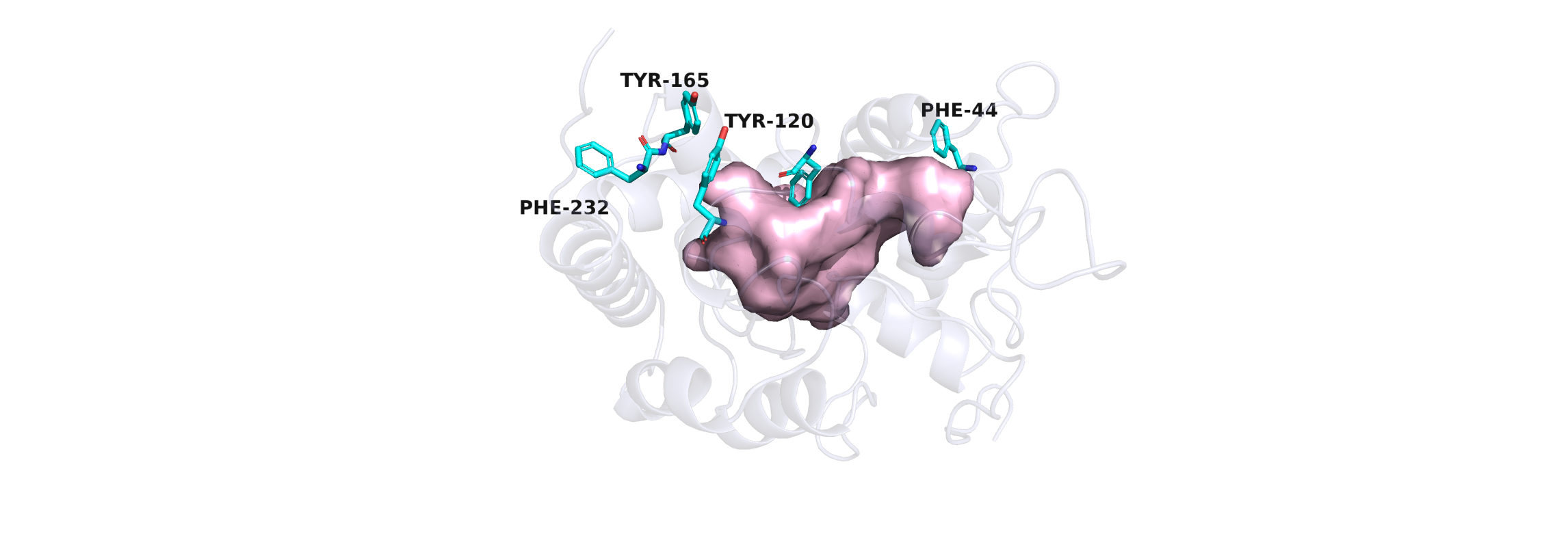 |
| *Sistotremastrum suecicum hhb10207ss3* | 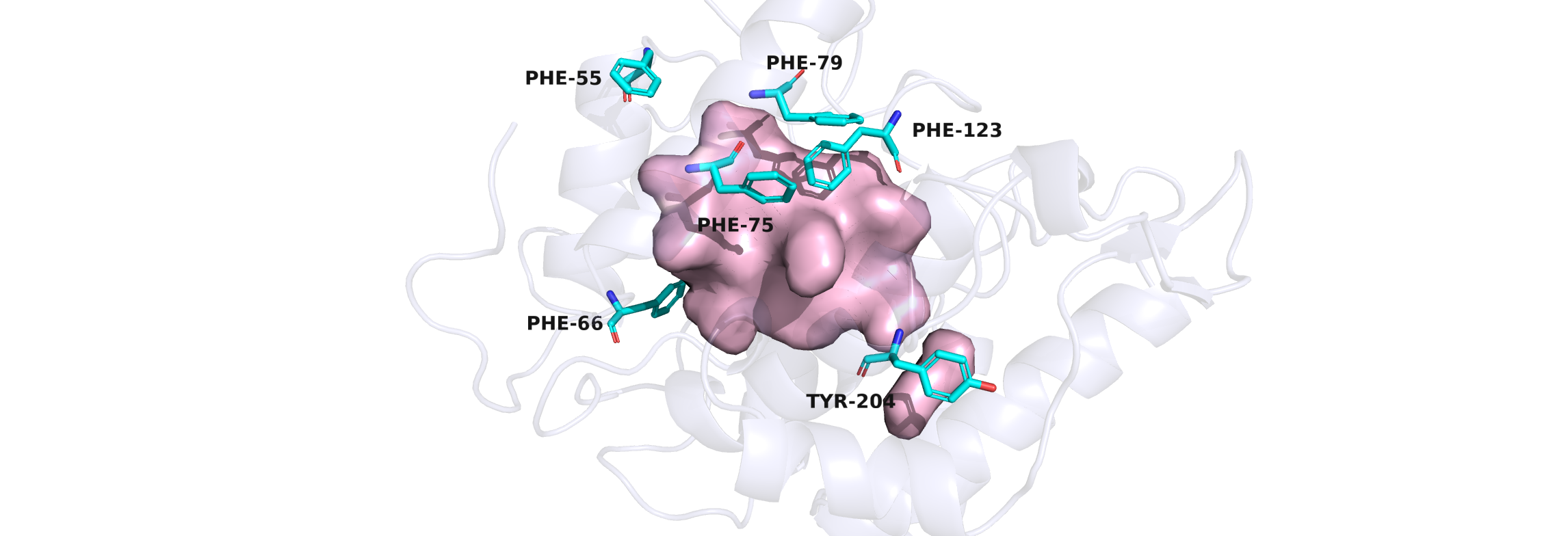 |
