# Supplementary figures and images for: New insights on unspecific peroxygenases: superfamily reclassification and evolution
Source: BMC Evol Biol. 2019 Mar 13;19:76. doi: 10.1186/s12862-019-1394-3 (PMC6417270; doi:10.1186/s12862-019-1394-3)

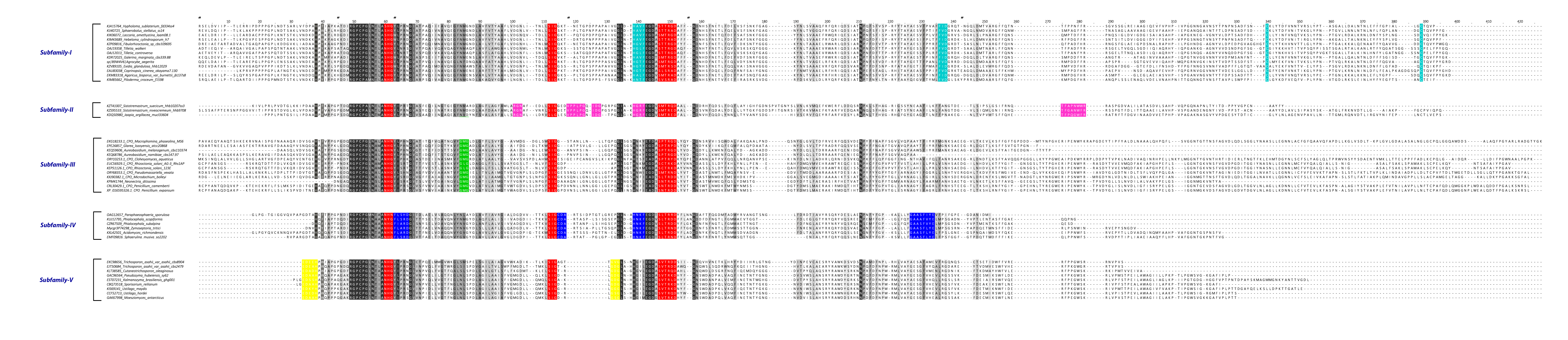

Supplement: Supplementary file 4 — Figure S2 MSA of the five different subfamilies of UPOs and newly found motifs highlighted with rectangles: sky blue for Subfamily-I, pink for Subfamily-II, green for Subfamily-III, blue represents motifs in Subfamily-IV, yellow represents motifs in Subfamily-V and red for the motifs found in all UPOs. (TIF 27409 kb) [file 12862_2019_1394_MOESM4_ESM.tif]

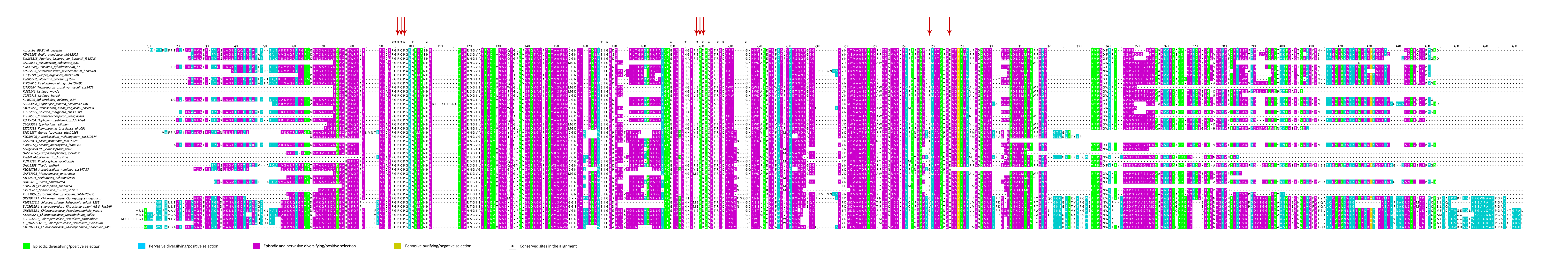

Supplement: Supplementary file 7 — Figure S3 MSA of all UPOs showing the positive and negatively selected sites using the MEME and FUBAR method. The asterisks (*) represent the conserved sites and the arrows point towards the motifs: PCP-EGD-R---E. (TIF 25856 kb) [file 12862_2019_1394_MOESM7_ESM.tif]

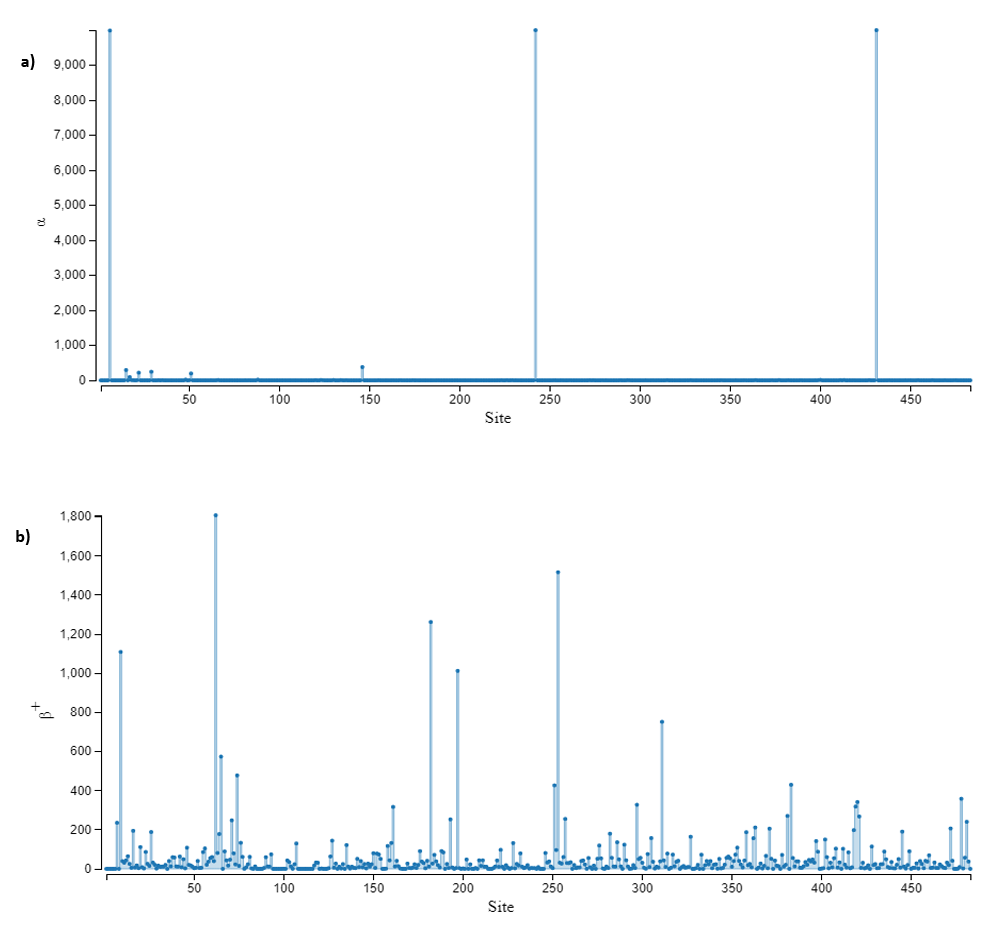

Supplement: Supplementary file 8 — Figure S4 A graph showing the number of (a) synonymous and (b) nonsynonymous sites in UPOs obtained using the MEME method. (TIF 649 kb) [file 12862_2019_1394_MOESM8_ESM.tif]

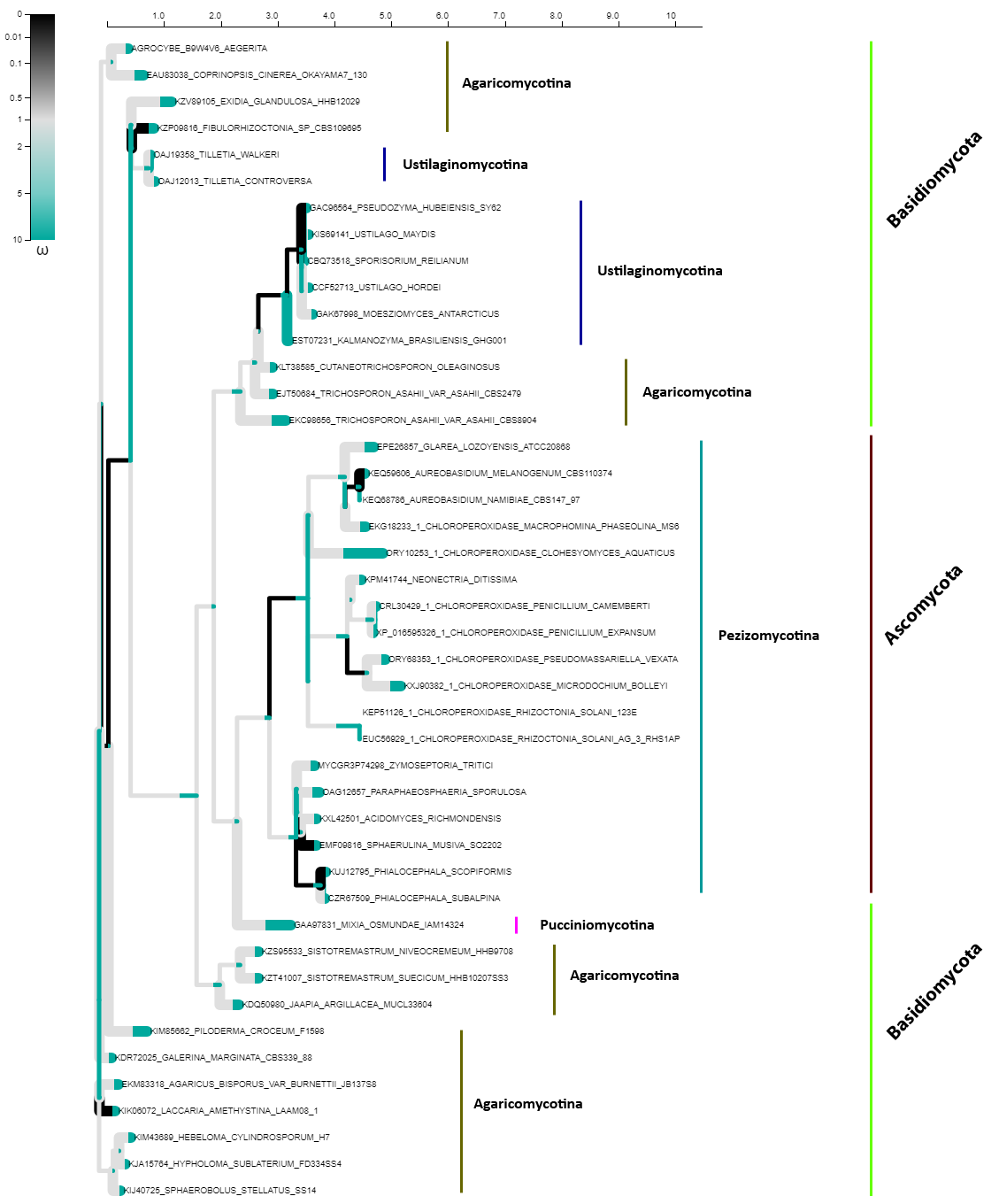

Supplement: Supplementary file 9 — Figure S5 Selection analysis on UPOs using aBSREL, a branch-site model. Thicker branches have a p-value < 0.05 showing evidence of undergoing positive diversifying selection. (TIF 1225 kb) [file 12862_2019_1394_MOESM9_ESM.tif]

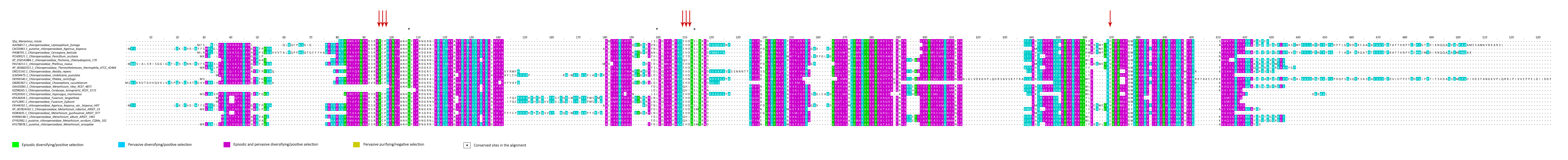

Supplement: Supplementary file 10 — Figure S6 MSA of all CPOs showing the positive and negatively selected sites using the MEME and FUBAR method. The asterisks (*) represent the conserved sites and the arrows point towards the motifs: PCP-EHD---E. (TIF 17764 kb) [file 12862_2019_1394_MOESM10_ESM.tif]

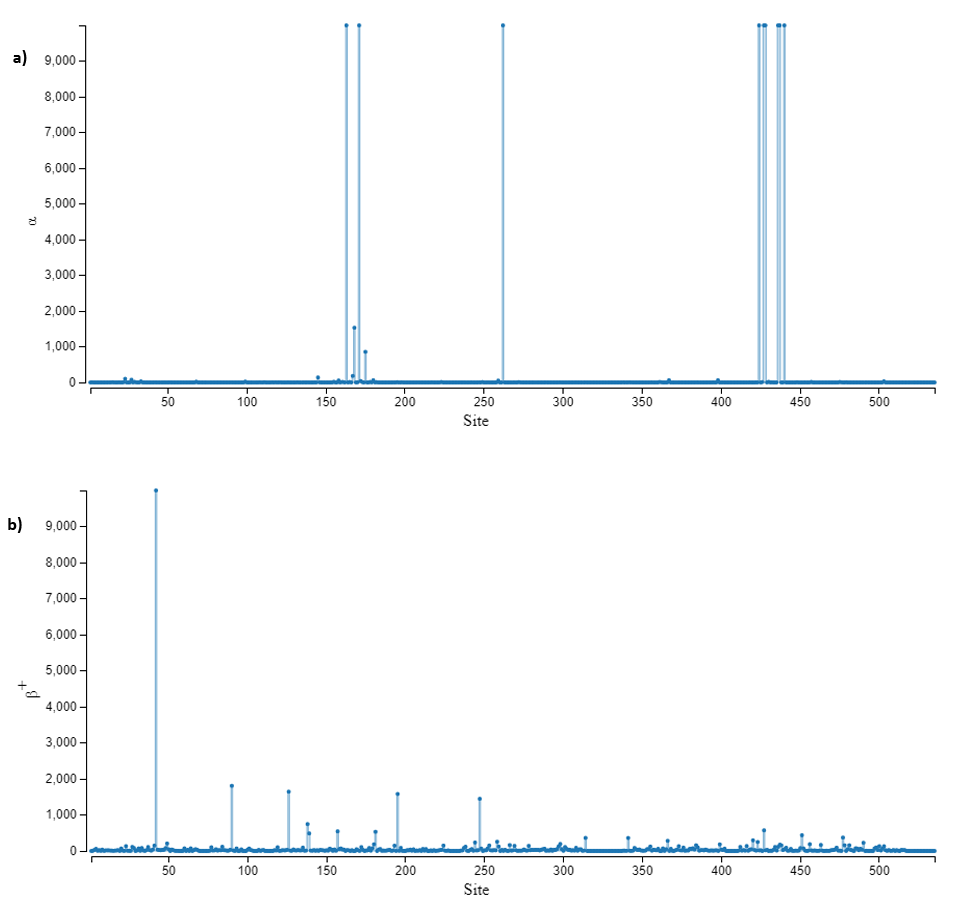

Supplement: Supplementary file 11 — Figure S7 A graph showing the number of (a) synonymous and (b) nonsynonymous sites in CPOs obtained using the MEME method. (TIF 602 kb) [file 12862_2019_1394_MOESM11_ESM.tif]

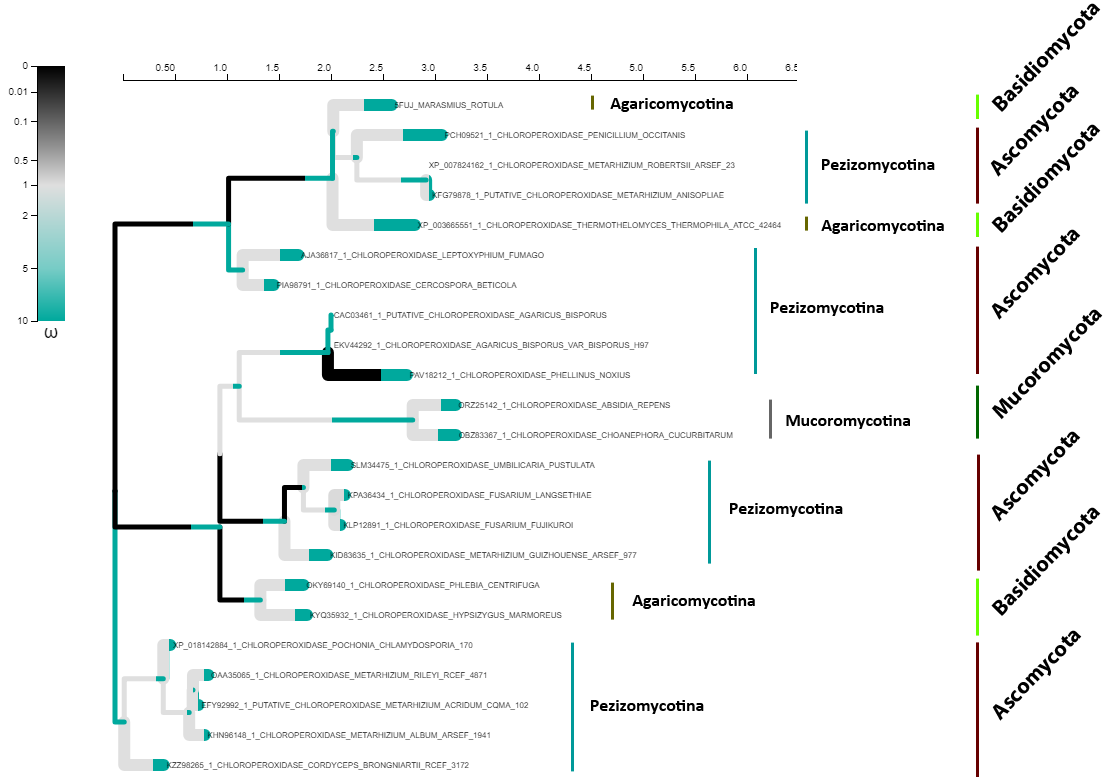

Supplement: Supplementary file 12 — Figure S8 Selection analysis on CPOs using aBSREL, a branch-site model. Thicker branches have a p-value < 0.05 showing evidence of positive diversifying selection. (TIF 1002 kb) [file 12862_2019_1394_MOESM12_ESM.tif]

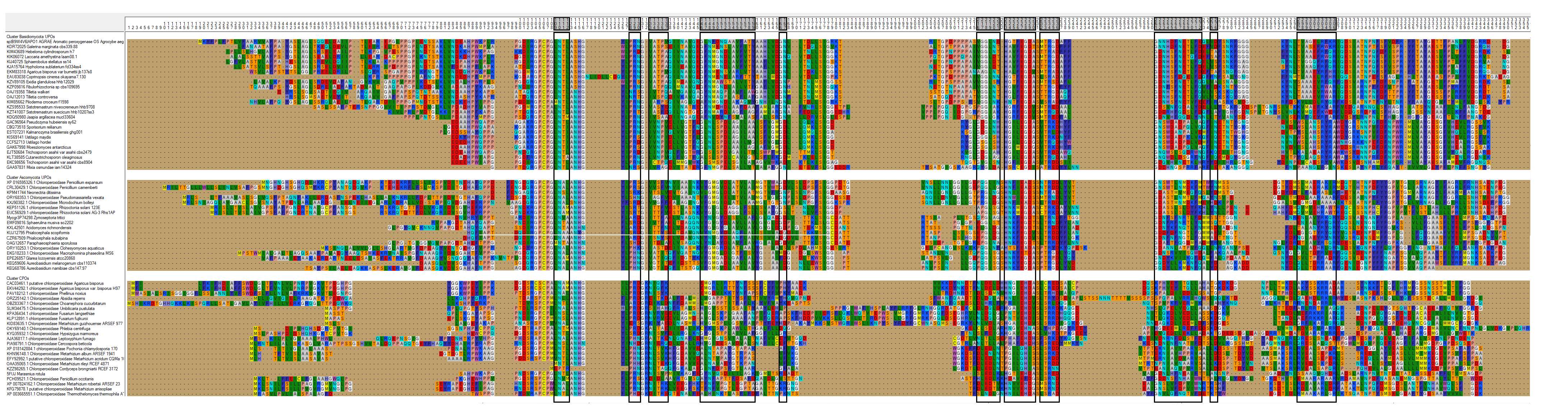

Supplement: Supplementary file 13 — Figure S9 An MSA of the clusters formed for the functional divergence analysis showing the Type-I functional divergent sites highlighted with black color. (TIF 7611 kb) [file 12862_2019_1394_MOESM13_ESM.tif]

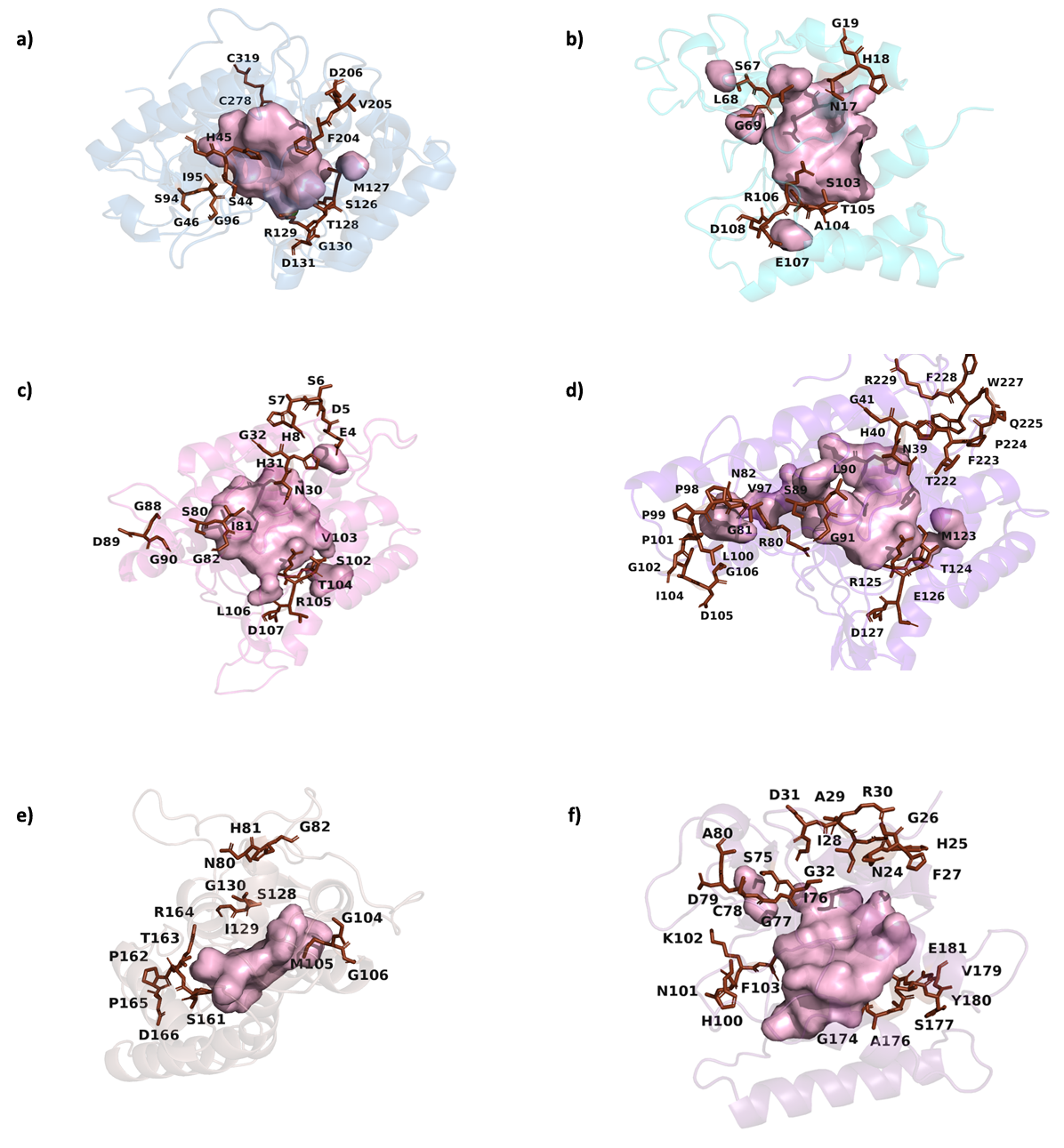

Supplement: Supplementary file 15 — Figure S10 Structural representation of the newly found motifs adhering near the binding pockets (shown as surface) are shown in one species from each subfamily of UPOs; a) experimentally resolved structure of AaeUPO and modeled structures of b) Mixia osmundae iam14324, c) Jaapia argillacea mucl33604, d) Kalmanozyma brasiliensis ghg001, e) Glarea lozoyensis atcc20868, and f) Phialocephala scopiformis. (TIF 2498 kb) [file 12862_2019_1394_MOESM15_ESM.tif]
