# Supplementary material for: New insights on unspecific peroxygenases: superfamily reclassification and evolution
Source: BMC Evol Biol. 2019 Mar 13;19:76. doi: 10.1186/s12862-019-1394-3 (PMC6417270; doi:10.1186/s12862-019-1394-3)
Supplement: Supplementary file 17 — Table S4 CPO sequences and MroUPO used in this study. (DOCX 14 kb) [file 12862_2019_1394_MOESM17_ESM.docx]

| Accession Number | Organism name | No. of amino acids |
| --- | --- | --- |
| AJA36817.1 | *Leptoxyphium fumago* | 376 |
| CAC03461.1 | *Agaricus bisporus* | 284 |
| PIA98791.1 | *Cercospora beticola* | 378 |
| PCH09521.1 | *Penicillium occitanis* | 257 |
| XP_018142884.1 | *Pochonia chlamydosporia 170* | 264 |
| PAV18212.1 | *Phellinus noxius* | 308 |
| XP_016595326.1 | *Penicillium expansum* | 421 |
| XP_003665551.1 | *Thermothelomyces thermophila ATCC 42464* | 261 |
| ORZ25142.1 | *Absidia repens* | 298 |
| ORY68353.1 | *Pseudomassariella vexata* | 441 |
| ORY10253.1 | *Clohesyomyces aquaticus* | 413 |
| SLM34475.1 | *Umbilicaria pustulata* | 262 |
| OKY69140.1 | *Phlebia centrifuga* | 404 |
| OBZ83367.1 | *Choanephora cucurbitarum* | 268 |
| OAA35065.1 | *Metarhizium rileyi RCEF 4871* | 227 |
| KZZ98265.1 | *Cordyceps brongniartii RCEF 3172* | 206 |
| KXJ90382.1 | *Microdochium bolleyi* | 439 |
| KYQ35932.1 | *Hypsizygus marmoreus* | 265 |
| KPA36434.1 | *Fusarium langsethiae* | 269 |
| KNZ75620.1 | *Termitomyces sp. J132* | 289 |
| CRL30429.1 | *Penicillium camemberti* | 446 |
| KLP12891.1 | *Fusarium fujikuroi* | 270 |
| EKV44292.1 | *Agaricus bisporus var. bisporus H97* | 284 |
| XP_007824162.1 | *Metarhizium robertsii ARSEF 23* | 267 |
| KID83635.1 | *Metarhizium guizhouense ARSEF 977* | 265 |
| KHN96148.1 | *Metarhizium album ARSEF 1941* | 269 |
| KEP51126.1 | *Rhizoctonia solani 123E* | 393 |
| EUC56929.1 | *Rhizoctonia solani AG-3 Rhs1AP* | 393 |
| EKG18233.1 | *Macrophomina phaseolina MS6* | 424 |
| EFY92992.1 | *Metarhizium acridum CQMa 102* | 266 |
| KFG79878.1 | *Metarhizium anisopliae* | 267 |
| 5FUJ | *Marasmius rotula* | 236 |

**Additional Table S4** CPO sequences and *Mro*UPO used in this study.
